# Synthetic glycans control gut microbiome structure and mitigate colitis in mice

**DOI:** 10.1038/s41467-022-28856-x

**Published:** 2022-03-10

**Authors:** Andrew C. Tolonen, Nicholas Beauchemin, Charlie Bayne, Lingyao Li, Jie Tan, Jackson Lee, Brian M. Meehan, Jeffrey Meisner, Yves Millet, Gabrielle LeBlanc, Robert Kottler, Erdmann Rapp, Chris Murphy, Peter J. Turnbaugh, Geoffrey von Maltzahn, Christopher M. Liu, Johan E. T. van Hylckama Vlieg

**Affiliations:** 1 Kaleido Biosciences, Lexington, MA 02421 USA; 2 glyXera GmbH, 39120 Magdeburg, Germany; 3 Max Planck Institute for Dynamics of Complex Technical Systems, 39106 Magdeburg, Germany; 4 Department of Microbiology and Immunology, University of California San Francisco, San Francisco, CA 94143 USA; 5 Flagship Pioneering, Cambridge, MA 02142 USA; 6 Present Address: Seres Therapeutics, Cambridge, MA 02139 USA; 7 Present Address: Pareto Bio, Cambridge, MA 02140 USA; 8 Present Address: Bacainn Therapeutics, Inc and Morningside BioPharma Advisory, Concord, MA 01742 USA; 9 Present Address: Exo Therapeutics, Watertown, MA 02472 USA

**Keywords:** Biopolymers, Microbiome

## Abstract

Relative abundances of bacterial species in the gut microbiome have been linked to many diseases. Species of gut bacteria are ecologically differentiated by their abilities to metabolize different glycans, making glycan delivery a powerful way to alter the microbiome to promote health. Here, we study the properties and therapeutic potential of chemically diverse synthetic glycans (SGs). Fermentation of SGs by gut microbiome cultures results in compound-specific shifts in taxonomic and metabolite profiles not observed with reference glycans, including prebiotics. Model enteric pathogens grow poorly on most SGs, potentially increasing their safety for at-risk populations. SGs increase survival, reduce weight loss, and improve clinical scores in mouse models of colitis. Synthetic glycans are thus a promising modality to improve health through selective changes to the gut microbiome.

## Abstract (web summary)

Here, the authors characterize the gut microbiome fermentation properties and therapeutic potential of chemically diverse synthetic glycans (SGs), showing they promote specific shifts in taxonomic and metabolite profiles, and exhibit therapeutic benefits in mouse models of colonic inflammation, together implying SGs as a potential avenue to treat disease by modulating the composition and metabolites produced by the gut microbiome.

## Introduction

Gut microbiome composition and metabolic output have been associated with initiation and progression of diseases including auto-immune diseases, cancer, metabolic syndrome, and liver disease^1–5^. Targeted manipulation of the microbiome is thus a promising therapeutic strategy being pursued by numerous public and private initiatives to treat disease^6^. In particular, glycan intervention is a chemical-based approach to alter the microbiome that leverages how ecological niches in the gut are occupied by species that are specialized to metabolize different carbon sources^7,8^. Alimentary interventions with complex glycans comprising dietary fiber significantly shift the composition and output of the gut microbiome within a few days and have been linked to prevention of type 2 diabetes, obesity, and cancer^9–12^.

Glycans with documented health benefits currently marketed as prebiotics include fructo-oligosaccharides (FOS), galacto-oligosaccharides (GOS), xylo-oligosaccharides (XOS), pullulan, and lactulose^13–17^. These glycans are extracted from agricultural materials or enzymatically synthesized and lack the structural complexity of dietary fiber, which is composed of a matrix of β-glucans, starches, hemicelluloses, and pectins with diverse monosaccharides and glycosidic bonds. To exploit the potential of glycans to modulate the microbiome, there is a need for novel molecules spanning the chemical and structural diversity of dietary glycans that can be efficiently and consistently produced. By controlling the starting materials and reaction conditions, we synthesized a library of hundreds of synthetic glycans (SGs) with different monosaccharides, bond types, and degrees of polymerization (DPs)^18,19^. These SGs enable a wide range of targeted changes to the microbiome and potentially open new avenues for the prevention and treatment of disease.

**Fig. 1:**
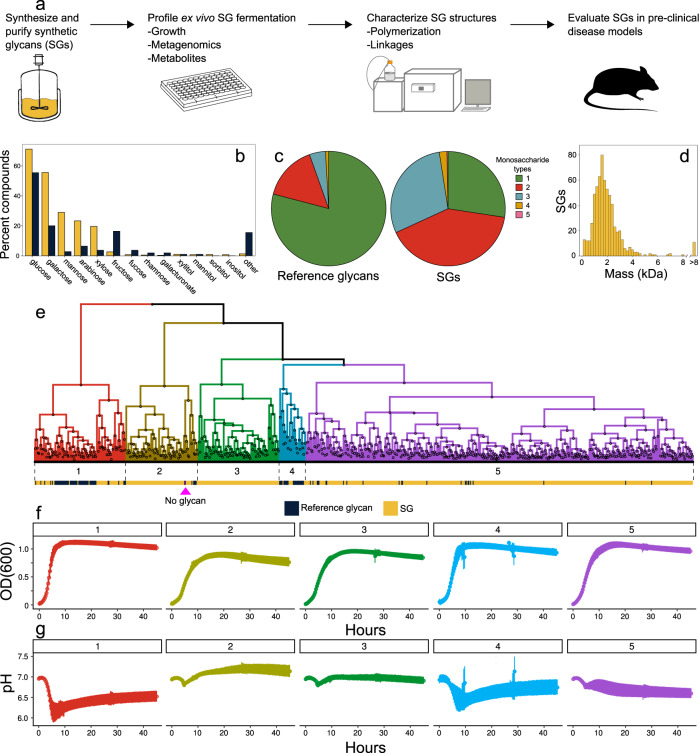
Analytical pipeline and description of glycan compositions and fermentation dynamics. **a** Schematic representation of the analytical pipeline. **b**–**f** Monosaccharide compositions and fermentation dynamics of 653 SGs and 110 reference glycans. **b** Percentages of SGs (yellow) and reference glycans (indigo) containing various monosaccharide types. **c** Number of monosaccharide types composing each SG or reference glycan. **d** Distribution of weight average molecular weights of SGs measured by SEC. **e**–**g** Growth (OD_600_) and pH dynamics of triplicate fecal cultures fermenting 5 g l^−1^ of a single SG or reference glycan in MM29 medium. **e** Hierarchical clustering of glycans into five fermentation groups based on twelve growth and pH parameters. Bars below the dendrogram show compound class: SG (yellow), reference glycan (indigo), or no glycan (magenta). Mean (**f**) growth and (**g**) pH curves (±SD) for each glycan fermentation group shown in **e**. Source data are provided as a Source Data file. SGs Synthetic Glycans, SEC size exclusion chromatography, OD_600_ optical density at 600 nm, SD standard deviation, kDa kilodalton.

In this study, the fermentation of hundreds of SGs polymerized from diverse, naturally occurring monosaccharides are compared to reference glycans using an ex vivo platform for highly multiplexed measurements of growth, metabolic output, and taxonomy. Based on the ex vivo assays, we select SGs for chemical structural analyses of their polymerization profiles and glycosidic linkages. As some reference glycans have been shown to alleviate intestinal inflammation and improve barrier function, as well as reduce colitis from infection, we compare the effects of SG and reference glycan treatment in mouse models of these intestinal pathologies^20,21^. Together, this experimental pipeline (Fig. 1a) evaluates the function and therapeutic potential of SGs as microbiome modulators and demonstrates additional benefits of SGs compared to reference glycans, including compounds currently marketed as prebiotics.

## Results

### SGs vary in composition and fermentation dynamics

We selected a set of 653 SGs and 110 commercially available, reference glycans that are compositionally diverse and sufficiently soluble for culture-based growth and metabolite assays (Supplementary Data 1, Supplementary Table 1). Both SGs and reference glycans are composed of a similar set of monosaccharides (Fig. 1b), but unlike the reference glycans, most SGs (73%) contain multiple, different monosaccharides (Fig. 1c), demonstrating how SGs can be built to include dietary sugars in novel and complex combinations. SGs were catalytically synthesized^19^ and span a wide range of average molecular masses (Fig. 1d) with a median of 1.7 kDa (range 0.3 kDa-77.5 kDa), corresponding to a polymerization of approximately ten monosaccharides. We profiled these SGs and reference glycans in a panel of ex vivo assays and highlighted the performance of two SGs with different compositions, BRF (glucose) and BQM (galactose and glucose), which were subsequently selected for structural analysis and mouse models.

We investigated variation in fermentation dynamics across the 763 glycans by measuring growth and pH kinetics of anaerobic fecal cultures. Hierarchical clustering of growth and pH parameters (Fig. 1e, Supplementary Fig. 1) identified groups of glycans with distinct growth (Fig. 1f) and pH (Fig. 1g) profiles. Reference glycans were enriched in group 1 (Fisher exact test *p* = 7.5 × 10^−26^) and group 4 (Fisher exact test *p* = 4.8 × 10^−14^), which both supported rapid growth resulting in a precipitous pH drop. Group 1 included pullulan, lactulose, GOS, and FOS; group 4 included XOS. Group 5, which included BQM and BRF, was enriched in SGs (*p* = 6.0 × 10^−17^) that were well-fermented at controlled rates with gradual reductions in pH. Thus, SG composition affects fermentability and SGs are generally fermented more slowly than reference glycans, likely due to their compositional complexity (Fig. 1c). In addition, these fermentation parameters were well correlated between fecal samples from two different donors (Supplementary Fig. 1c–n). While more data is needed to establish that SG effects are conserved across populations, these data suggest SGs have similar fermentation dynamics between individuals.

### SGs change microbiome metabolic output

**Fig. 2:**
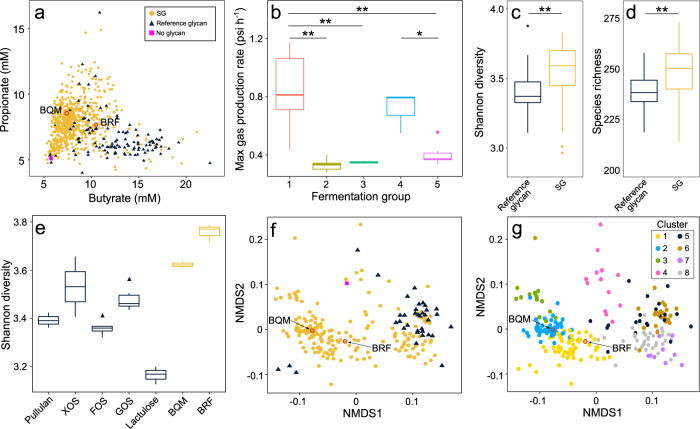
Effects of glycans on fecal community metabolic output and taxonomic composition. **a** Yields of two SCFAs, butyrate and propionate, from fecal cultures fermenting either an SG (yellow circles, *n* = 653), reference glycan (indigo triangles, *n* = 110), or no glycan (magenta square). **b** Maximum gas production rate (psi h^−1^) during fecal culture fermentation of glycans from each of the five fermentation groups in Fig. 1e–g. **c** Shannon diversity and **d** species richness of fecal cultures fermenting SGs (yellow, *n* = 190) versus reference glycans (indigo, *n* = 40). **e** Shannon diversity of fecal cultures fermenting BRF or BQM (yellow) is higher than reference glycans (indigo) for all comparisons except BQM versus XOS (Kruskal–Wallis followed by Dunn’s comparison test, *p* < 0.05). **f** NMDS of metagenomic data calculated based on a matrix of Bray–Curtis dissimilarities using species-level mapping of sequencing reads from fecal cultures grown on either an SG (yellow circles, *n* = 190), reference glycan (indigo triangles, *n* = 40), or no glycan (magenta square). **g** NMDS as in **f** colored by differences in taxonomic composition defined by eight K-means clusters based on species-level mapping of sequencing reads. Data for each glycan is the mean of (**a**–**d**, **f**, **g**) three or (**e**) six replicate fecal cultures grown on 5 g l^−1^ of each SG or reference glycan for 45 h in MM29 medium. **a**, **f**, **g** BRF and BQM highlighted in red. **b**–**e** Box plots show median and interquartile ranges. Asterisks show significance (*p < 0.05, **p < 0.01) by **b** Tukey’s test or **c**, **d** two-sided Wilcoxon rank-sum test. Source data are provided as a Source Data file. SG Synthetic Glycan, SCFA short-chain fatty acid, XOS xylo-oligosaccharides, FOS fructo-oligosaccharides, GOS galacto-oligosaccharides, NMDS non-metric multidimensional scaling.

Fermentation of glycans by the gut microbiome produces metabolites including short-chain fatty acids (SCFAs) that have important physiological and immunological benefits^15,22^. We developed a high-throughput matrix-assisted laser desorption/ionization time-of-flight (MALDI-TOF) mass spectrometry method to quantify yields of two SCFAs, butyrate and propionate, in fecal cultures fermenting each of the 763 glycans. Butyrate is the preferred energy source of colonocytes, leading to healthy colonocyte function and maintenance of an anaerobic gut environment^23^. Propionate has immunological and metabolic effects locally in the gut and is absorbed into the bloodstream to regulate cholesterol^24,25^. In addition, butyrate and propionate promote differentiation of naive T cells into anti-inflammatory regulatory T cells^26,27^. Butyrate and propionate production by some SGs were similar to those of reference glycans, but many SGs were distinct in producing high propionate levels (Fig. 2a), supporting these SGs favor growth of propionate-producing taxa such as *Bacteroidaceae* and *Ruminococcaceae*^25^.

We investigated the extent to which SCFA production underlies pH dynamics during glycan fermentation by measuring a time series of three main SCFAs (acetate, propionate, and butyrate) by gas chromatography in cultures fermenting a subset of glycans. The correlation between pH and production of these SCFAs was weak, but the relative levels of SCFAs and ammonia, an abundant microbial product, were strongly correlated with pH (Supplementary Fig. 2). While these SCFA measurements did not include lactate because it is non-volatile and can not be quantified by standard gas chromatography^28^, our data supports that changes in pH observed during glycan fermentation are associated with the balance between SCFA and ammonia production.

In addition to SCFAs, glycan fermentation produces gaseous compounds that can lead to abdominal bloating, a major symptom limiting tolerability of reference glycans in patients with gastrointestinal disorders such as irritable bowel syndrome^29^. As the volume and rate of gas produced depend on diet and microbiome composition^30^, we measured gas production rates by fecal communities growing on BRF, BQM, and randomly selected glycans from each of the five fermentation groups in Fig. 1e–g (Supplementary Data 2). The rate of gas production varied widely among groups (Fig. 2b) and reflected fermentation dynamics. Glycans in fermentation groups 1 and 4, mostly reference glycans that are rapidly fermented, had the most rapid gas production. SGs in group 5, which included BRF and BQM, produced gas more moderately, potentially improving tolerability in humans.

### SGs alter microbiome taxonomic composition

Glycan fermentation can result in divergent changes to microbiome composition, even differentially promoting closely related species^8^. Therefore, we applied shotgun metagenomic sequencing to examine fecal cultures fermenting a compositionally diverse set of 190 SGs and 40 reference glycans (Supplementary Data 3). While there is heterogeneity in the level of microbiome diversity resulting from fermentation of different SGs, SG fermentation generally resulted in higher taxonomic diversity (Fig. 2c) and species richness (Fig. 2d) relative to reference glycans, as shown by BQM and BRF promoting higher diversity relative to reference glycans (Fig. 2e).

Fecal community compositions following SG fermentations spanned the taxonomic space covered by reference glycan fermentations, but also reached novel compositions (Fig. 2f) that can be explored by clustering based on taxa abundances. SG-enriched taxa clusters had elevated abundances of *Lachnospiraceae* (clusters 1–3) and *Parabacteroides* (clusters 2–3) (Fig. 2g, Supplementary Fig. 3a–c). BRF is in glycan cluster 1 (Fig. 2g, Supplementary Fig. 3a) with increased abundances of members of the family *Lachnospiraceae* including *Roseburia*, a key butyrate producer that is reduced in the microbiomes of patients with Crohn’s disease^31^, and *Fusicatenibacter*, which is decreased in patients with active ulcerative colitis^32^. BQM is in glycan cluster 2 (Fig. 2g, Supplementary Fig. 3b) with elevated levels of other *Clostridiaceae* and *Parabacteroides*, taxa associated with remission in Crohn’s disease^33^. Clusters enriched in reference glycan fermentations contained elevated levels of *Lactobacillaceae* (cluster 5) and *Bifidobacterium* (clusters 6–8) (Fig. 2g, Supplementary Fig. 3e–h), which are adapted to metabolize simple oligosaccharides such as FOS^34^.

### SGs are poor growth substrates for model pathogens

**Fig. 3:**
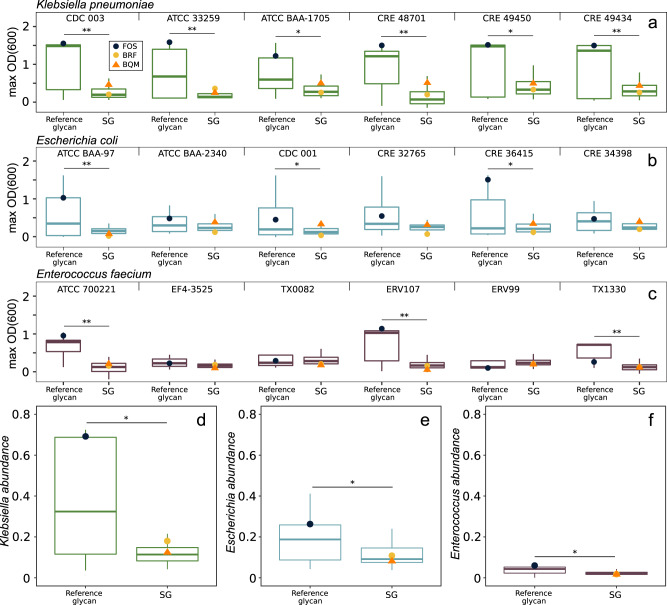
Enteric pathogen growth in pure culture and relative abundances in fecal communities grown on glycans. Six strains of **a** *Klebsiella pneumoniae*, **b** *Escherichia coli*, or **c** *Enterococcus faecium* were cultured with 5 g l^−1^ of a single SG (*n* = 148) or reference glycan (*n* = 32) in CM3 medium. Data is the mean maximum growth (OD_600_) of triplicate cultures; strain names are shown above each plot. Fecal communities from a healthy donor were OD_600_-normalized to contain 8% of **d** *K. pneumoniae* CDC 003, **e** *E. coli* CDC 001, or **f** *E. faecium* ATCC 700221 and cultured in triplicate with 5 g l^−1^ of an SG (*n* = 45) or reference glycan (*n* = 17) for 45 h in MM29 medium. The relative abundances of the pathogens were quantified by 16S rRNA gene sequencing. Data points show FOS (indigo circle), BRF (yellow circle), or BQM (orange triangle) cultures. Box plots show median and interquartile ranges. Asterisks show significance (*p < 0.05, **p < 0.01) by two-sided Wilcoxon rank-sum test. Source data are provided as a Source Data file. SGs Synthetic Glycans, OD_600_ optical density at 600 nm, FOS fructo-oligosaccharides.

It is important to establish that microbiome-modulating glycans do not promote proliferation of pathogens. In particular, intestinal colonization by a multidrug-resistant (MDR) pathogen such as carbapenem-resistant *Enterobacteriaceae* (CRE) expressing extended-spectrum carbapenemases or vancomycin-resistant *Enterococcus* (VRE) is a major risk factor for bloodstream infection^35,36^. Currently, no approved therapies exist for de-colonization of these pathogens from the gut. We hypothesized that SGs will confer a competitive growth advantage to polysaccharide-utilizing commensals relative to pathogens in gut microbiomes^37^. Initially, we examined the ability of a panel of laboratory and MDR clinical *Enterobacteriaceae* and *Enterococcus* strains (Supplementary Table 2) to grow on each of a compositionally diverse set of 148 SGs including BRF and BQM and 32 reference glycans including FOS (Supplementary Data 4) as a sole carbohydrate source in defined medium. Most strains of the model enteric pathogens *Klebsiella pneumoniae* (Fig. 3a), *Escherichia coli* (Fig. 3b), and *Enterococcus faecium* (Fig. 3c) exhibited limited growth on SGs with final cell densities significantly lower than when grown on reference glycans including FOS.

To assess changes in the relative abundances of model pathogens in gut microbiomes during glycan fermentation, we spiked a healthy donor fecal community with either CRE *K. pneumoniae*, CRE *E. coli*, or VRE *E. faecium*, cultured it in a medium containing each SG or reference glycan (Supplementary Data 5), and measured changes in relative abundances of the pathogen by 16S rRNA gene sequencing. The relative abundances of *K. pneumonia* (Fig. 3d), *E. coli* (Fig. 3e), and *E. faecium* (Fig. 3f) were significantly lower after growth on SGs including BRF and BQM than on reference glycans, consistent with the SGs favoring growth of commensal taxa.

In contrast to the limited growth of pathogens on SGs relative to reference glycans, pure cultures of phylogenetically diverse, gut commensals (Firmicutes, Bacteroidetes, and Actinobacteria) grown in defined medium often reached similar cell densities with either BRF, BQM, or glucose as the sole carbohydrate source (Supplementary Fig. 4a–f). We measured a time series of BRF and BQM consumption by a healthy donor fecal community using high-performance anion exchange chromatography with pulsed amperometric detection (HPAEC-PAD), which showed the fecal community fully consumed these SGs by progressively metabolizing fractions of increasing DP (Supplementary Fig. 5).

### SGs reproducibly shift microbiome taxonomic and CAZyme profiles across individuals

Based upon the assimilated data from the ex vivo experiments, we focused on BRF and BQM because they have different monosaccharide compositions, are well-fermented, enrich different, anti-inflammatory taxa associated with resolution of colitis, promote high taxonomic diversity, and are poor growth substrates for pathogen-associated genera. To gain insight into how these SGs alter microbiome taxonomy and function, we assessed the effects of treatment with BRF, BQM, or lactulose on fecal communities from additional healthy donors by metagenomics (ten donors for SGs, seven donors for lactulose). Lactulose was included as a reference glycan that is hydrolyzed and metabolized by the colonic microbiota^38^. Lactulose is, to our knowledge, the only medically prescribed glycan that acts primarily through modulation of the microbiome and is commonly used to prevent and treat hepatic encephalopathy^39^.

**Fig. 4:**
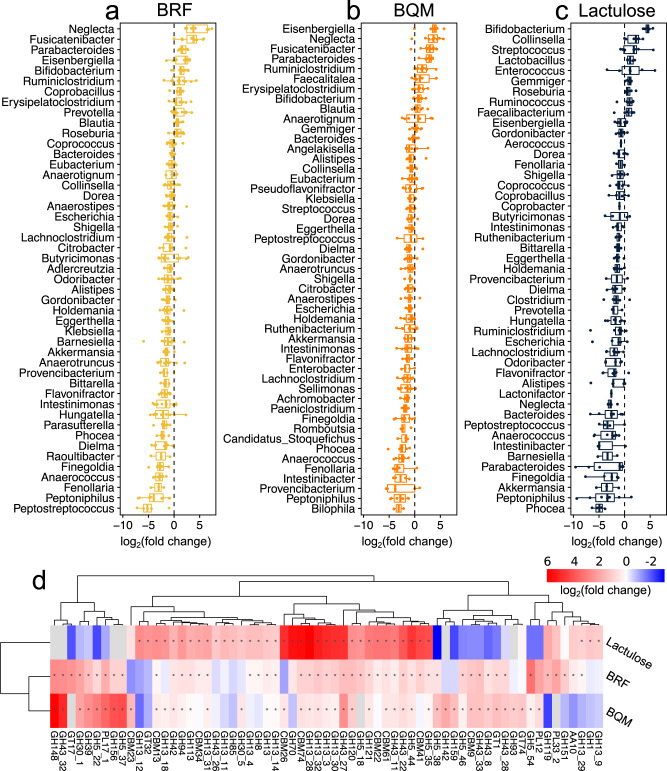
Changes in fecal community composition in response to glycans across human donors. Differences in relative abundances of genera in ex vivo fecal cultures grown on (**a**) BRF, (**b**) BQM, or (**c**) lactulose relative to no-glycan controls. Data points show median log_2_FC in genus abundances for each donor; box plots show median and interquartile range across donors. Genera with significant changes (*p* < 0.05 after FDR correction) are shown. **d** Differences in CAZyme gene abundances in ex vivo cultures grown on different glycans (median log_2_FC versus no-glycan controls). CAZyme family/subfamilies with significant abundance changes (*p* < 0.05 after FDR correction) and log_2_FC > 1 on at least one glycan versus no-glycan controls are shown with hierarchical clustering based on Euclidean distance. Colors are FC with gray showing families not detected in the no-glycan control. Asterisks indicate significantly elevated abundance. **a**–**d** Fecal cultures from healthy donors (ten donors for SGs, seven donors for lactulose) were grown in triplicate for 45 h in MM29 medium supplemented with 5 g l^−1^ glycan, as appropriate, and sequenced by metagenomics. Statistical significance for genus and CAZyme changes was determined by fitting a linear mixed-effect model on rank transformed genera/CAZyme abundance data with glycan treatment as fixed effect and subject as random effect. Source data are provided as a Source Data file. SGs Synthetic Glycans, FC fold change, FDR false discovery rate, CAZyme carbohydrate-active enzyme, GH glycoside hydrolase, CBM carbohydrate-binding modul, PL polysaccharide lyase, GT glycosyltransferase, AA auxiliary activity.

Ex vivo fecal metagenomics revealed glycan treatments resulted in taxonomic shifts that were conserved across individuals (Fig. 4a–c). Both SGs promoted *Parabacteroides* along with Clostridia such as *Ruminiclostridium* and multiple genera of *Lachnospiraceae*. There were also differences in taxa promoted by each SG. For example, *Roseburia* and *Prevotella* abundance were specifically elevated in the BRF treatment (Fig. 4a), similar to metagenomics of the BRF-containing glycan cluster 1 (Supplementary Fig. 3a). In contrast, lactulose treatment increased the relative abundance of *Bifidobacterium* and *Lactobacillus*, similar to the metagenomics of glycan cluster 5 that includes lactulose (Supplementary Fig. 3e).

Glycan treatment was also associated with widespread changes in the abundances of carbohydrate-active enzyme (CAZyme) genes in fecal metagenomes (Fig. 4d). Metabolism of an oligosaccharide glycan requires specific CAZymes to cleave its glycosidic linkages^40,41^, leading to promotion of the subset of taxa in the microbiome encoding relevant CAZymes^42,43^. As such, the abundances of genes for CAZymes that are active on an oligosaccharide should increase in metagenomes following treatment. The abundances of glycoside hydrolases (GHs) known to cleave lactulose such as GH42^44^ were most highly elevated in the lactulose cohort, along with thirteen GH13 subfamilies (Fig. 4d). GH13 are not known to cleave lactulose, but are widespread in the genomes of Bifidobacteria^45^, which increased in response to lactulose (Fig. 4c). Both SGs resulted in increased abundances of GH148 (β-1,3-glucanases) along with six subfamilies of GH5 containing predicted β-glucosidases and β-glucanases that are likely active on the glucose linkages present in BRF and BQM. In addition, the metagenomes of the BQM-treated communities were enriched in galactanase CAZyme families such as GH159 (β-D-galactofuranosidase)^46^ and GH43 (7 subfamilies) that putatively cleave the galactose linkages in BQM (Fig. 4d).

### SG structural features

**Fig. 5:**
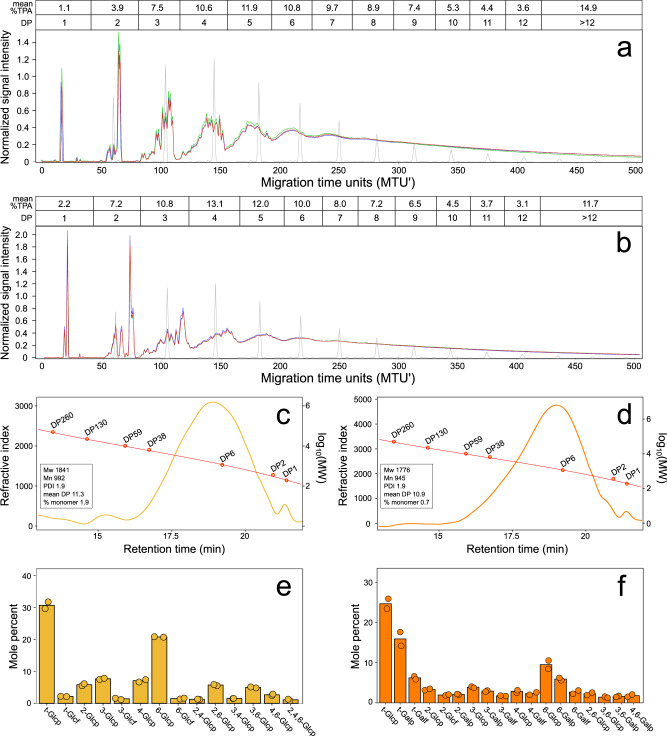
Structural features of SGs. xCGE-LIF analysis of the DP of (**a**) BRF and (**b**) BQM. Triplicate measurements of each SG (red, green, purple) and oligo-maltose standards (gray) are shown relative to normalized migration time units (MTU’). The mean percent TPA at each DP is above the plots. SEC chromatograms of (**c**) BRF and (**d**) BQM showing distributions relative to molecular weight (MW) standards with insets showing polymerization parameters. Refractive index in millivolts and molecular weights in Daltons. **e**–**f** Abundances (mole percent) of monosaccharides with different glycosidic linkages in (**e**) BRF and (**f**) BQM. Labels show all linkage types at >1% mole percent with residues linked only at 1- position as terminal “t-” residues. Bars show means and points show measurements for independent syntheses of each glycan. Source data are provided as a Source Data file. SGs Synthetic Glycans, xCGE-LIF multiplexed capillary gel electrophoresis with laser-induced fluorescence detection, DP degree of polymerization, TPA total peak area, SEC size exclusion chromatography, Mw weight average molecular weight, Mn number average molecular weight, PDI polydispersity index, Glcp glucopyranose, Glcf glucofuranose, Galp galactopyranose, Galf galactofuranose.

We performed a detailed characterization of the chemical structures of BRF and BQM. The chemical catalysis methods for synthesis of BRF and BQM (see Methods) yielded an ensemble of structures that were defined as a function of monosaccharide input, catalyst, and reaction conditions^19^. Multiplexed capillary gel electrophoresis with laser-induced fluorescence detection (xCGE-LIF) showed BRF (Fig. 5a) and BQM (Fig. 5b) have low monomer content (1.1% and 2.2%, respectively). We observed a series of discrete peaks indicating multiple structures at each DP below five, but the increased structural complexities at higher DPs exceeded resolution. Size exclusion chromatography of BRF (Fig. 5c) and BQM (Fig. 5d) confirmed their low monomer contents and showed similar average DPs of 11.3 and 10.9, respectively. Both compounds have a polydispersity index of 1.9 due to the range of molecular masses comprising each SG.

We performed linkage analysis of BRF (Fig. 5e) and BQM (Fig. 5f) to examine the abundances and types of glycosidic bonds. Both glycans contained mixtures of 1,2- 1,3-, 1,4-, and 1,6-bonds with frequent branching, as >2 linkages were present in 19% of monosaccharide subunits in BRF and 14% of subunits in BQM. We further compared the glycosidic linkages of BRF and BQM relative to reference glycans by two-dimensional nuclear magnetic resonance spectroscopy (2D-NMR). The 2D-NMR profiles of BRF versus pullulan (Supplementary Fig. 6a) and BQM versus GOS (Supplementary Fig. 6b) support that these SGs contain a greater diversity of glycosidic bonds with distinct stereo- and regiochemistries. The structural complexity of SGs likely contributes to their comparatively slow fermentation by commensals (Fig. 1f, g).

### SGs demonstrate therapeutic potential in mouse models

**Fig. 6:**
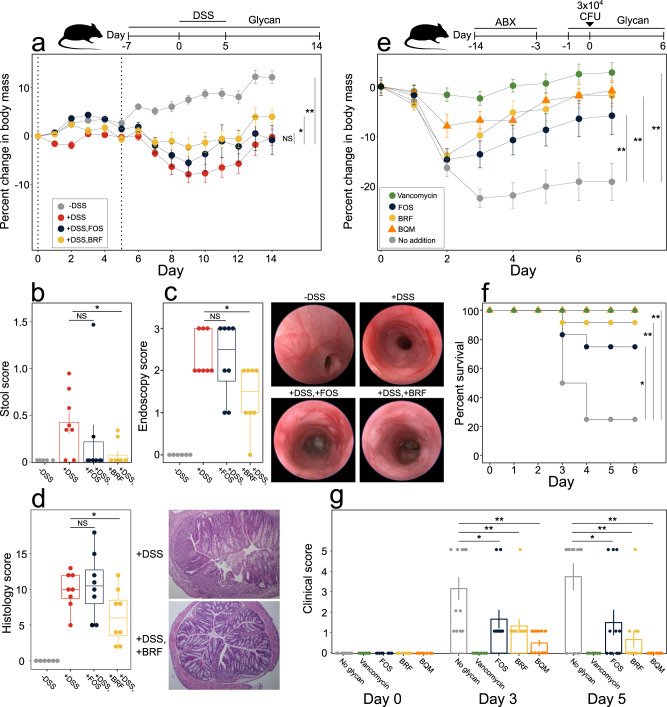
Glycan effects in mouse models of DSS colitis and *C. difficile infection*. **a**–**d** Mice were treated in drinking water with 2.5% DSS (days 0–5, dashed lines) and 1% (v/v) glycans (days 7–14), as appropriate. Treatment groups (eight animals per group): −DSS (gray), +DSS (red), +DSS, FOS (indigo), +DSS, BRF (yellow). Treatment group comparisons of (**a**) body weight, (**b**) stool score averaged over days 0–14, (**c**) day 14 endoscopy scores with representative images, and (**d**) day 14 histology scores with representative 100x magnified H&E stained distal colon micrographs. **e**–**g** Mice were treated with antibiotics (days -14–3), infected with *C. difficile* (day 0), and treated with 50 mg kg^−1^ vancomycin daily (days 0–4) or 1% (v/v) glycans in drinking water (days 1–6), as appropriate. Treatment groups (12 animals per group): no glycan (gray), vancomycin (green), FOS (indigo), BRF (yellow circles), and BQM (orange triangles). Treatment group comparisons of (**e**) body weight, (**f**) survival, and (**g**) clinical scores. Data in (**a**, **b**, **e**, **g**) show treatment group means ± SEM. Box plots in (**c**, **d**) show median and interquartile range. Data points in (**b**–**d**, **g**) show individual mice. **a**, **e** Statistics on body mass changes are based on area under the curve for all individual mice. Asterisks show significance (*p < 0.05, **p < 0.01) by (**a**–**e**, **g**) two-sided Wilcoxon rank-sum test or (**f**) log-rank test. Source data are provided as a Source Data file. DSS dextran sodium sulfate, FOS fructo-oligosaccharides, ABX antibiotics, CFU colony forming units, SEM standard error of the mean, NS non-significant.

We evaluated the therapeutic potential of BRF and BQM in mouse models of intestinal damage and disease. Initially, we examined the effects of BRF or FOS in a mouse model of dextran sodium sulfate (DSS)-induced colitis (Fig. 6a). BRF treatment reduced DSS-mediated weight loss (Fig. 6a) and improved scores of diarrhea (Fig. 6b) and endoscopy (Fig. 6c). Colonic histology showed DSS induced lesions including epithelial erosion or mucosal ulceration, loss of colonic glands, and inflammation of the mucosa, which was reduced by BRF treatment relative to DSS alone (Fig. 6d). In contrast, FOS treatment failed to reduce weight loss or improve scores of stool, endoscopy, or histology relative to DSS treatment without glycan supplementation (Fig. 6a–d).

We also examined if BRF and BQM could alleviate pathologies associated with *C. difficile* infection. First, we confirmed that *C. difficile* grows poorly on BRF or BQM as a sole carbohydrate source in defined medium (Supplementary Fig. 4g). We then tested if BRF, BQM, or FOS have in vivo efficacy in a *C. difficile* murine infection model (Fig. 6e). Fecal metagenomics on day 6 after *C. difficile* infection revealed a divergence in fecal microbiome compositions across treatments (Supplementary Fig. 7a) with the abundances of different genera elevated in each glycan treatment (Supplementary Fig. 7b–e). Further, we found these microbiome changes translated into improved outcomes. Glycan treatment significantly reduced weight loss (Fig. 6e) and increased survival (Fig. 6f) relative to the no-glycan treatment. Whereas 3 of 12 mice survived in the no-glycan treatment, 11 of 12 mice survived BRF treatment and BQM treatment had 100% survival, similar to the vancomycin-treated controls (Fig. 6f). In addition, all glycans improved clinical scores relative to the no-glycan treatment (Fig. 6g). While FOS improved all metrics relative to the no-glycan treatment, BQM further reduced weight loss (Fig. 6e), increased survivorship (Fig. 6f), and improved clinical scores (Fig. 6g) relative to FOS.

## Discussion

Perturbations to the taxonomic composition and metabolic output of the gut microbiome have been linked to numerous non-communicable and immune-related pathologies. Delivery of rationally optimized, complex glycans is a promising option to drive the composition of the microbiota toward health-promoting states by leveraging taxa-specific differences in glycan metabolism, one of the dominant factors shaping gut microbiome composition^7^. Our study demonstrates SGs are a novel, glycan-based modality to manipulate the properties of human gut communities with flexibility and precision. SGs are synthetic compounds with mixtures of glycosidic bonds that recapitulate features of the chemical complexity of dietary fiber, while avoiding challenges associated with natural fibers including geographic and seasonal variability of raw material sourcing. Moreover, SGs are well-metabolized by polysaccharide-utilizing commensals, but not important pathogens. While still requiring validation in human trials, SGs could represent an antibiotic-sparing approach to control infection with no known mechanism of resistance.

Both of the SGs that we tested in mouse models shifted fecal community composition to elevate potentially anti-inflammatory taxa that are negatively associated with inflammatory bowel disease (IBD) including *Lachnospiraceae* (especially *Roseburia* and *Fusicatenibacter*), *Ruminiclostridium*, and *Parabacteroides*^31–33,47^. As such, the SGs BQM and BRF could potentially alleviate altered microbiome compositions associated with mild and moderate inflammation, which is supported by how these SGs lessen pathologies associated with DSS colitis (Fig. 6a–d) and *C. difficile* infection (Fig. 6e–g) in mice. SG-mediated microbiome shifts could also be beneficial for other diseases. *Roseburia* is reduced in the microbiomes of colorectal cancer patients^48^*. Fusicatenibacter* and *Parabacteroides* are proposed to promote resistance to *C. difficile* infection^49,50^ and *Parabacteroides* is associated with decreased hepatic steatosis^51^.

SGs extend the benefits of reference glycans and avoid many of the limitations of microbiome interventions based on live bacteria including dosing, engraftment, and inconsistent manufacturing. SGs could also be applied to promote growth and engraftment of live bacteria therapeutics. SGs selected for clinical development can be produced using controlled manufacturing processes and have been evaluated as Generally Regarded as Safe (GRAS) for their intended use in clinical research, allowing generation of proof of mechanism information by streamlined, human interventions. Together, our results show SGs are chemically diverse glycans that effectuate novel taxonomic and metabolic shifts to gut communities and reduce symptoms of colitis in mouse models. Future work will further expand the potential of SGs in disease management by modulating the composition and metabolites produced by the gut microbiome.

## Methods

### Synthesis of BRF and BQM

BRF was synthesized by combining D-( + )-glucose (100.0 g, 555.1 mmol), Dowex® Marathon™ C (7.0 g, 5% dry weight ratio to glucose, 29% moisture content), and 30.0 mL DI water in a 1000 mL 3-necked round bottom flask equipped with an overhead stirrer, thermocouple plug, and a short-path distillation head. The mixture was stirred continuously at 100 rpm using a glass stirring shaft equipped with a Teflon half-moon paddle. The reaction mixture was run at 130 °C for 4 h. To quench the reaction, 60 mL DI water was added to the reaction mixture. The Dowex resin was removed by vacuum filtration through a fritted-glass filter. The solution was then diluted to 25.0 °Bx and purified by precipitation. The solution was slowly poured into absolute ethanol to form a cloudy solution with a final water:ethanol ratio of 1:9 (v/v). The cloudy solution was then centrifuged at 2100 × *g* for 2 h. The supernatant was removed, and the precipitate was collected and dissolved in water. The residual ethanol was removed under reduced pressure. The solution was frozen at −20 °C and lyophilized to yield the final product of white powder (60.5 g, 61% yield).

BQM was synthesized by combining D-( + )-glucose (50.0 g, 277.5 mmol), D-( + )-galactose (50.0 g, 277.5 mmol), Dowex® Marathon™ C (7.0 g, 5% dry weight ratio to monosaccharides, 29% moisture content), and 30.0 mL DI water in a 1000 mL 3-necked round bottom flask equipped with an overhead stirrer, thermocouple plug, and a short-path distillation head. The mixture was stirred continuously at 100 rpm using a glass stirring shaft equipped with a Teflon half-moon paddle. The reaction mixture was run at 130 °C for 4 h. To quench the reaction, 60 mL DI water was added to the reaction mixture. The Dowex resin was removed by vacuum filtration through a fritted-glass filter. The solution was then diluted to 25.0 °Bx and purified by precipitation. The solution was slowly poured into absolute ethanol to form a cloudy solution with a final water:ethanol ratio = 1:9 (v/v). The cloudy solution was then centrifuged at 2100 x *g* for 2 h. The supernatant was removed, and the precipitate was collected and dissolved in water. The residual ethanol was removed under reduced pressure. The solution was frozen at −20 °C and lyophilized to yield the final product of white powder (57.2 g, 57% yield).

### Size exclusion chromatography (SEC)

Glycan samples (300 mg) were resuspended in 10 mL water, 0.2 mm filtered, and 10 μL of glycan solution was injected to an Agilent 1100 HPLC system with refractive index (RI) detector equipped with a guard column (Agilent PL aquagel-OH, 7.5 × 50 mm, 5 µm, PL1149-1530), and two SEC columns (2X Agilent PL aquagel-OH 20,PL1120-6520) in tandem connection. The sample was run with 0.1 M NaNO_3_ mobile phase, 28 min run time, 0.9 mL min^−1^ flow rate with the column and RI detector at 40 °C. The peaks of the sample were integrated and the weight-average molecular mass (M_w_), number average molecular mass (M_n_), mean degree of polymerization (mean DP), and polydispersity index (PDI) were determined using Agilent Cirrus GPC/SEC software (version 3.4.2). The calibration curve was generated from polymer standard solutions (10 mg mL^−1^) of D-( + ) Glucose Mp 180, Carbosynth Ltd Standard; Maltose Mp 342, Carbosynth Ltd Standard; Maltohexaose Mp 990, Carbosynth Ltd Standard; Nominal Mp 6100 Pullulan Standard, PSS # PPS-pul6k; Nominal Mp 9600 Pullulan Standard, PSS # PPS-pul10k; Nominal Mp 22000 Pullulan Standard, PSS # PPSpul22k; and Nominal Mp 43000 Pullulan Standard, PSS # PPSpul43k.

### xCGE-LIF oligosaccharide analysis

The DP distribution of SGs were determined using a glyXboxCE™ (glyXera GmbH, Magdeburg, Germany) based on multiplexed capillary gel electrophoresis with laser-induced fluorescence detection (xCGE-LIF)^52^. SG samples were prepared using a glyXprep kit for oligosaccharide analysis (KIT-glyX-OS.P-APTS-48-01, glyXera). Briefly, samples were diluted 100-fold with ultrapure water and labeled with fluorescent dye 8-aminopyrene-1,3,6-trisulfonic acid (APTS). Two µL of each SG sample, 2 µL APTS Labeling Solution, and 2 µL ReduX Solution were mixed thoroughly and incubated for 3 h at 37 °C. The labeling reaction was stopped by adding 150 µL Stopping Solution and the excess of unreacted APTS and salt were removed using glyXbeads for hydrophilic interaction chromatography solid phase extraction (HILIC-SPE). The samples were aliquoted to wells containing 200 µL glyXbead slurry and incubated for 5 min at ambient temperature for binding, followed by washing and elution steps.

The purified APTS-labeled glycans were analyzed on a glyXboxCE™ system (glyXera) equipped with a 50 cm 4-capillary array, filled with POP-7™ polymer (4333466 and 4363929, Thermo Fisher Scientific). The sample (1 µL) was mixed with 1 µL prediluted 1^st^ NormMiX (STD-glyX-1stN-100Rn-01, glyXera)^53^ internal standard to align migration times for migration time unit (MTU’) calculations. The mixture was combined with 9 µL glyXinject (C-glyXinj-1.6mL-01, glyXera) and subjected to xCGE-LIF analysis. The samples were electrokinetically injected and measured with a running voltage of 15 kV for 40 min. Data were analyzed with glyXtoolCE™ (glyXera) glycoanalysis software to perform migration time alignment, raw data smoothing, DP-range specific interval picking (summing up of the total measuring signal within the DP ranges after comparison with a reference oligo-maltose ladder), and normalization of total peak areas.

### Glycan linkage analysis

Permethylation was performed as previously described with slight modification^54^. The glycan sample was purified to a monosaccharide content <1.0% using chromatography before permethylation. The glycan sample (500 µg) was dissolved in dimethyl sulfoxide (DMSO) for 30 min with gentle stirring. A freshly prepared sodium hydroxide suspension in DMSO was added, followed by a 10 min incubation. Iodomethane (100 µL) was added, followed by a 20 min incubation. A repeated round of sodium hydroxide and iodomethane treatment was performed for complete permethylation. The permethylated sample was extracted, washed with dichloromethane (DCM), and blow dried with nitrogen gas. The sample was hydrolyzed in 2 M trifluoroacetic acid (TFA) for 2 h, reduced overnight with sodium borodeuteride (10 mg mL^−1^ in 1 M ammonia), and acetylated using acetic anhydride/TFA. The derivatized material was extracted, washed with DCM, and concentrated to 200 µL. Glycosyl linkage analysis was performed on an Agilent 7890 A GC equipped with a 5975 C MSD detector (EI mode with 70 eV), using a 30-meter RESTEK RTX®-2330 capillary column. The GC temperature program: 80 °C for 2 min, a ramp of 30 °C min^−1^ to 170 °C, a ramp of 4 °C min^−1^ to 245 °C, and a final holding time of 5 min. The helium flow rate was 1 mL min^−1^, and the sample injection was 1 µL with a split ratio of 10:1.

### 2D HSQC NMR

Structures of SGs were compared to reference glycans: pullulan (P4516 Sigma) or galacto-oligosaccharides (DOMO Vivinal GOS). Glycans were lyophilized and a 20 mg sample was dissolved in 200 μL of deuterium oxide (D_2_O) with 0.1% (v/v) acetone as the internal standard. The solutions were placed into 3 mm NMR tubes and HSQC (heteronuclear single quantum coherence) spectra were recorded at 25 °C on a Bruker AVANCE III 500 MHz spectrometer equipped with a 5 mm BBF-H-D-05 probe with *Z*-axis gradient using the Bruker program “HSQCETGP.hires2”. HSQC experiments were performed with eight scans and a 1 s recycle delay. Each spectrum was acquired from 7.5–1.5 ppm in F2 (^1^H) with 1024 data points and 120–50 ppm in F1 (^13^C) with 256 data points. The resulting spectra were analyzed using the MestReNova software (version: 12.0.0-20080) from Mestrelab Research.

### Glycan consumption analysis

Consumption of SGs during fermentation by fecal cultures was measured at ProDigest (Ghent, Belgium) by high-performance anion exchange chromatography with pulsed amperometric detection (HPAEC-PAD) using an ICS-3000 chromatography (Dionex, Sunnyvale, CA, USA) equipped with a CarboPacPA20 column (Dionex)^55^. Sample preparation involved initial dilution of the sample with ultrapure water followed by deproteinization with acetonitrile (1:1), centrifugation (21,380 × *g*, 10 min), and filtration (0.2 μm PTFE, 13 mm syringe filter, VWR International) prior to injection (5 μL) into the column. Qualitative glycan fingerprints were obtained by plotting the detected signal (nC) against the elution time.

### Human fecal sample collection

Informed consent was obtained from all donors before fecal sample collection. To collect samples for ex vivo cultivation, donors were instructed to collect the bowel movement into a sample collection unit, which was immediately sealed and placed on ice. Within 4 h of bowel movement, sample collection units were transferred into an anaerobic chamber, unsealed, and processed into fecal slurries. To prepare fecal slurries for culturing, samples were transferred into filtered blender bags (Interscience) and diluted using 1x phosphate-buffered saline (PBS) and glycerol to result in 20% fecal slurry (w/w) containing 15% glycerol (w/w). Diluted samples were homogenized in a lab blender (Interscience 032230), flash frozen in a dry ice/ethanol bath, and stored at −80 °C.

Fecal samples from four donors were selected for ex vivo cultivations as representative samples from among a set of 32 donors by assessing the homogeneity of donors at basal conditions by metagenomic sequencing (Supplementary Fig. 8). The donor 1 sample was used for fermentation dynamics, metabolite analysis, gas production, and SG consumption analysis. The donor 2 sample was used for fermentation dynamics comparisons. The donor 3 sample was used for glycan culture metagenomics. The donor 4 sample was used for model pathogen spike-ins. In addition, the reproducibility of fecal community responses to glycan treatment across individuals was assessed by ex vivo cultivations of fecal samples from ten randomly selected healthy subjects for SGs (BRF, BQM) and seven healthy subjects for lactulose. These samples were not pre-screened by metagenomic sequencing to avoid biases in sample selection.

### Microbial cultivation

Fecal communities were cultured in Mega Medium 29 (MM29) (Supplementary Table 3) and single strains were grown in Clostridial Minimal 3 (CM3) medium (Supplementary Table 4). All cultures were grown anaerobically at 37 °C containing 5 g l^−1^ of the appropriate glycan as the sole carbohydrate source. Growth was measured in 60 μl cultures in 384 well plates (3860 Corning) sealed using a Breathe-Easy® sealing membrane (Z380059 Sigma). Cell densities at an optical density of 600 nm (OD_600_) and pH were measured using a Biotek Synergy H1 multi-mode plate reader outfitted with a Biostack 4 plate stacker. Fecal cultures were grown in MM29 medium for 45 h before DNA extraction for metagenomic or 16S rRNA gene sequencing. Gas production was measured continuously in 25 ml cultures fermenting different glycans in MM29 medium grown in 125 mL glass bottles sealed with an Ankom^*RF*^ gas production module (Ankom, Macedon NY, USA).

Pathogen growth in pure culture was measured for strains each of *K. pneumoniae, E. coli*, and *E. faecium* (Supplementary Table 2) cultured anaerobically in CM3 medium supplemented with 5 g l^−1^ of each glycan (Supplementary Data 4). To measure pathogen abundance in fecal cultures, fecal slurries were OD-normalized to contain 8% of each pathogen strain, grown for 45 h in MM29 medium supplemented with 5 g l^−1^ of each glycan (Supplementary Data 5), and relative abundances were quantified by 16S rRNA gene sequencing.

Culture pH was measured by supplementing the medium with 2 μM BCECF [2,7-bis-(2-carboxyethyl)-5-(and -6)-carboxyfluorescein] (C3411 Sigma) and the ratio of BCECF fluorescence at the pH-sensitive point (485 nm excitation; 540 nm emission) relative to the pH-insensitive isosbestic point (450 nm excitation; 540 nm emission) was measured^56^. The pH was calculated relative to a standard curve of media at known pH by fitting a sigmoidal curve using four parameter logistic regression. The R package, phgrofit (version 1.0.2)^57^, was developed to extract physiological descriptors from the kinetic pH and OD_600_ curves (Supplementary Fig. 1). Glycans were clustered based on twelve fermentation parameters that were transformed into *Z*-scores by subtracting the mean across all glycans and dividing by the standard deviation. Hierarchical clustering analysis on these *Z*-score values was performed using the Manhattan distance metric and the complete agglomeration method; cluster number was selected based on the “elbow method” to minimize the within cluster variation (sum of squares distance).

### DNA sequencing and analysis

Fecal microbiomes were characterized by 16S rRNA gene or shotgun metagenomic sequencing (Diversigen, MN USA). DNA was extracted from human and mouse fecal samples using Qiagen DNeasy PowerSoil extraction plates, quantified using the Quant-iT PicoGreen dsDNA assay (Thermo Fisher), and stored at −80 °C until analysis. 16S libraries were prepared by PCR amplification with the 515 F/806 R primer set and metagenomic libraries were prepared using the NexteraXT kit before sequencing on the Illumina platform. The average reads per sample for shotgun metagenomics was 0.5 million reads and for 16S metagenomics was 25 thousand reads.

Sequences of 16S rRNA genes were analyzed by UNOISE clustering^58^ and denoising of raw sequences followed by DADA2/RDP taxonomic calling at the genus or family level^59^. Taxa count tables from shotgun metagenomic sequencing data were generated by the SHOGUN pipeline^60^ using a database including the first 20 strains per species in RefSeq v87. Metagenomic reads with ambiguous species matches were excluded from taxa counts to reduce spurious matches.

To cluster SGs based on taxonomic response using metagenomics data from ex vivo *c*ultures, the relative abundance of each species was averaged across triplicate cultures for each glycan. *K*-means clustering was performed based on species relative abundances differences relative to no-glycan controls defined by species-level mapping of sequencing reads. The number of clusters (*K* = 8) was selected using the “elbow method” to minimize the within-cluster variation (sum of squares distance) while also minimizing *K*. For ecological analysis, relative abundance was averaged across technical replicates for each species. The *vegan* R package^61^ (version 2.5-6) was used to compute the Shannon diversity, Bray–Curtis dissimilarity, and multidimensional scaling measures and coordinates.

Sequencing reads corresponding to carbohydrate-active enzyme (CAZyme) genes in metagenomic data were identified based on alignment at 97% identity to a CAZyme gene sequence database, which includes all annotated CAZyme genes from all strains in the same database used for taxonomy assignment (see above). CAZyme genes in each strain were annotated using the Hidden Markov Models from dbCAN2^62^. Metagenomic reads were mapped to the CAZyme database using the BURST aligner^63^ to build a table of gene length-normalized count values for each CAZyme family/subfamily in a metagenomic sample.

### Short-chain fatty acid (SCFA) quantification

To quantify butyrate and propionate by matrix-assisted laser desorption/ionization-time of flight (MALDI-TOF) mass spectrometry, fecal culture supernatants and SCFA standards in culture medium were derivatized by 3-Nitrophenylhydrazine hydrochloride (3NPH, N21804 Sigma) through *N*-(3-Dimethylaminopropyl)-*N*′-ethylcarbodiimide hydrochloride (EDC, Sigma E6383) coupling^64^. Culture media (10 µL) was mixed with 10 µL of 100 mM EDC in 50:50 acetonitrile/water (v/v) containing 7.5% pyridine and 10 µL of 100 mM 3NPH in 50:50 acetonitrile/water (v/v), incubated at 45 °C for 1 h, and diluted with 30 µL of 50:50 acetonitrile/water (v/v). The matrix internal standard (IS) solution was prepared by derivatizing 25 mM of ^13^C-butyric acid and ^2^D-propionic acid using 3NPH and mixing with saturated 9-Aminoacridine (9AA, Sigma 92817) in 50:50 methanol/water (v/v) at a 1:4 volume ratio.

SCFA reaction mixtures were mixed 1:1 with matrix IS solution, co-crystallized onto a MALDI HTS target plate by a TTP Mosquito liquid handling system, and dried at room temperature. A Bruker Daltonics ultrafleXtreme MALDI-TOF/TOF mass spectrometer was used to acquire mass spectra in reflectron negative ion mode using 65% laser from laser attenuator and 2200-shots accumulation for each sample. Data was collected and processed using Bruker PharmaPulse 2.1 software. Peak area of derivatized butyrate (m/z 222.2) and propionate (m/z 208.2) were monitored and normalized with the peak area of corresponding internal standards (m/z 224.2 and 210.2, respectively). The concentrations of propionate and butyrate in each sample were calculated using calibration standards. Measurements of propionate and butyrate by MALDI-TOF and by gas chromatography with flame-ionization detection (GC-FID) were confirmed to be well correlated for ex vivo fecal cultures fermenting SGs (Supplementary Fig. 9).

To quantify acetate, butyrate, and propionate in fecal microbial culture supernatants by GC-FID, 50 μL of each sample and calibration standards (2.5, 10.0, 15.0, 20.0, 30.0, and 40.0 mM of each SCFA) were mixed with 20 μL of a 400 mM 2-ethylbutyric acid internal standard solution prepared in HPLC-grade water. Immediately before injection, 30 uL of each mixed sample was acidified with 100 µL of 6% formic acid and 1 μL was injected. The GC-FID (7890 A, Agilent) was run as follows: 15 m × 0.53 mm × 0.50 µm DB-FFAP column (Agilent); carrier gas was helium at 28.819 mL min^−1^; inlet conditions were 250 °C with a 5 mL min^−1^ purge flow at a 4:1 split ratio. The initial oven temperature of 70 °C was increased by 70 °C over 1 min, increased by 100 °C over 1 min, and held at 240 °C for 1.8 min. Chromatogram peaks of SCFA standards were used to derive a retention time window and quantification was based on plotting each standard against detector response (x) with a 1/x weighting factor.

### DSS colitis mouse model

All animal studies were performed in facilities accredited by the Association for Assessment and Accreditation of Laboratory Animal Care (AAALAC). Male C57Bl/6 mice (Taconic Biosciences) at 6–8 weeks of age were randomized into cages each containing a treatment group of eight mice at Biomodels LLC (Waltham MA, USA). Mice were kept in rooms provided with filtered air at 65–75 °F with 30–70% relative humidity. Rooms were on a 12 h:12 h light:dark cycle with no twilight and a minimum of 12–15 air changes per hour. Animals were fed diet 5053 (Lab Supply, Fort Worth TX, USA) throughout the study with water provided *ad libitum*. All animals were weighed daily and assessed visually for the presence of diarrhea and/or bloody stool. Mice with >30% weight loss were euthanized. Colitis was induced by supplementing the drinking water with 2.5% dextran sodium sulfate (DSS) from days 0–5. Glycans were administered in the drinking water as 1% (v/v) filter-sterilized solutions from days 7–14.

Stool consistency for each mouse was scored daily (Supplementary Table 5). Colitis in each mouse was assessed by video endoscopy under isoflurane anesthesia on day 14. During each endoscopic procedure, images were recorded and colitis severity was scored by a blinded observer (Supplementary Table 6). Histopathological changes in tissue samples were assessed by a board-certified veterinary pathologist at Inotiv (Missouri, USA). Tissue samples were prepared by fixation in formalin and longitudinal and cross-sections of proximal and distal colon were processed, embedded in paraffin, sectioned at ~5–6 μm, mounted on glass slides, stained with hematoxylin and eosin (H&E) examined microscopically, and scored using a qualitative and semi-quantitative grading system (Supplementary Table 7).

### *C. difficile* infection mouse model

Female C57Bl/6 mice (Harlan Laboratories) weighing 16–18 g were randomly allocated into treatment groups of 12 animals (3 animals per cage) at Trans Pharm Pre-clinical Solutions (Jackson MI, USA). Animal room conditions were the same as in the DSS colitis study. Mice were fed Teklad Global Rodent Diet 2918 (Harlan Laboratoires) and water *ad libitum*. All mice received an antibiotic cocktail (0.5 mg ml^−1^ kanamycin, 0.044 mg ml^−1^ gentamicin, 1062 U ml^−1^ colistin, 0.27 mg ml^−1^ metronidazole, 0.16 mg ml^−1^ ciprofloxacin, 0.1 mg ml^−1^ ampicillin, 0.06 mg ml^−1^ vancomycin, 1% (v/v) glucose) in their drinking water from day 14–5 followed by a single oral dose of 10 mg kg^−1^ clindamycin on day 3. On day 0, mice received an oral gavage of 3 × 10^4^ viable *C. difficile* spores (strain VPI 10463 ATCC 43255). Mice in the vancomycin control group received oral gavage of 50 mg kg^−1^ vancomycin daily from day 0–4. Glycans were administered in the drinking water as 1% (v/v) filter-sterilized solutions from day 1–6. Glycan efficacy was assessed by daily measurements of animal survival, body weight, and a clinical score of disease severity (Supplementary Table 8). Mouse feces were placed into a 96-well plate that was kept on wet ice and then frozen at −80 °C. Separate disposable forceps were used for each cage of animals to avoid cross-contamination.

### Statistical analysis

Statistical analyses were performed using R software. Box plots show median and interquartile ranges. Values are presented as the mean ± SD for ex vivo fermentation dynamics, bacterial growth, and glycan linkage analyses and as the mean ± SEM in mouse models. Comparisons of ex vivo cultures fermenting reference glycans versus SGs were evaluated by two-sided Wilcoxon rank-sum test for Shannon diversity, species richness, and enteric pathogen growth and relative abundances. Differences in Shannon diversity between cultures fermenting individual glycans were evaluated by Kruskal–Wallis followed by Dunn’s comparison test. Statistical significance for gas production experiments was performed by Tukey’s test after confirming the data adhered to a normal distribution by Shapiro Wilks test. Statistical significance for abundance changes in genera and CAZyme families across individuals was determined by fitting a linear mixed effect model on rank transformed genera/CAZyme abundance data with glycan treatment as fixed effect and subject as random effect, and corrected to a false discovery rate < 0.05.

In mouse models, animals were allocated randomly to each treatment group and different groups were processed identically. Body mass changes are based on the area under the curve for each individual mouse. Statistical significance of comparisons between no-glycan controls and other treatments for body mass, stool scores, endoscopy scores, and histology scores were evaluated by two-sided Wilcoxon rank-sum test. Differences in survivorship between no-glycan controls and other treatments were calculated by log-rank test.

### Reporting summary

Further information on research design is available in the Nature Research Reporting Summary linked to this article.

## Supplementary information

Supplementary Information

Description of Additional Supplementary Files

Supplementary Data 1

Supplementary Data 2

Supplementary Data 3

Supplementary Data 4

Supplementary Data 5

Reporting Summary

## Data Availability

The DNA sequencing reads for this study are available in the NCBI SRA database as project accessions PRJNA800419, PRJNA800763, PRJNA800792, and PRJNA800405. Supporting data are available in the Supplementary Information, Supplementary Data, and Source data file. All requests will be reviewed by Kaleido Biosciences to verify whether the request is subject to any intellectual property or confidentiality obligations. Source data are provided with this paper.

## Source data

Source Data

## Code availability

The methods and code for the R package phgrofit (version 1.0.2) to extract physiological descriptors from the kinetic pH and OD_600_ data have been deposited in Github and are freely available^57^.
