# Supplementary material for: Synthetic glycans control gut microbiome structure and mitigate colitis in mice
Source: Nat Commun. 2022 Mar 10;13:1244. doi: 10.1038/s41467-022-28856-x (PMC8913648; doi:10.1038/s41467-022-28856-x)
Supplement: Supplementary file 1 — Supplementary Information [file 41467_2022_28856_MOESM1_ESM.pdf]

Supplementary Information for “Synthetic glycans control gut microbiome structure and mitigate colitis in mice”

Supplementary Figures 1-9

Supplementary Tables 1-8

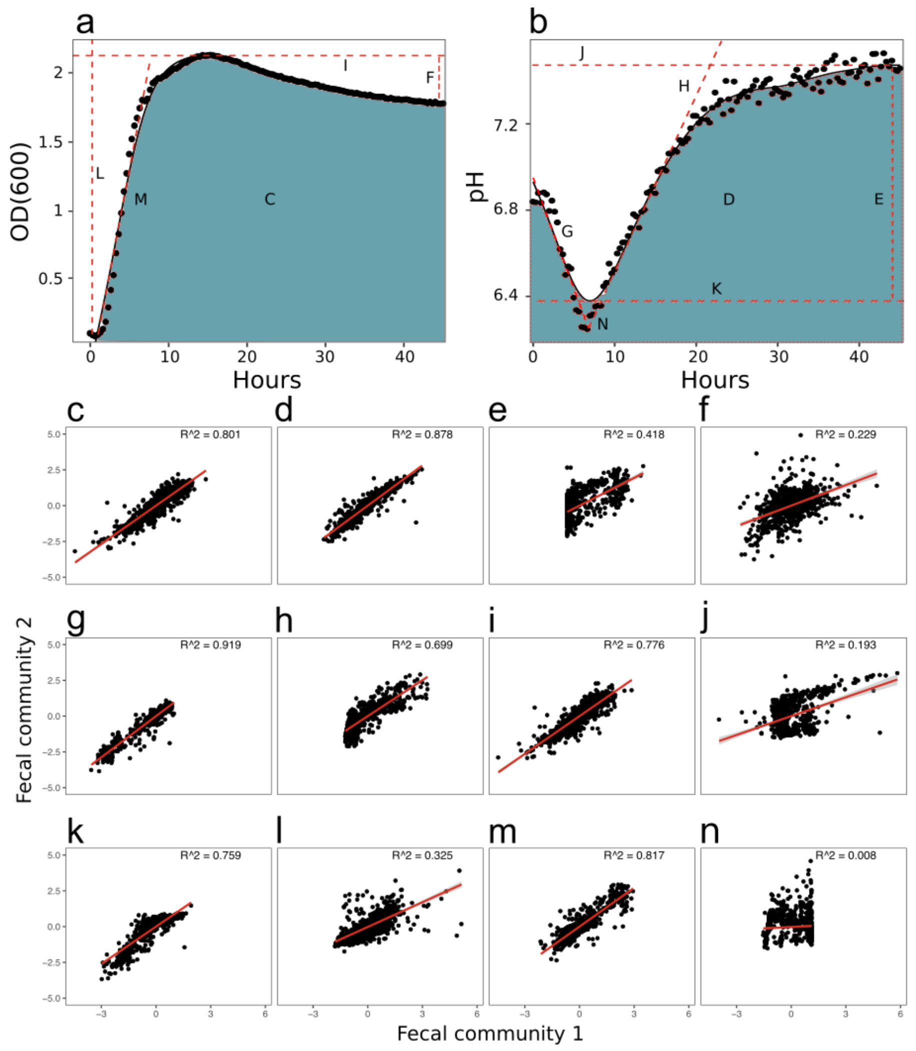

Supplementary Fig 1 Method to measure glycan fermentation dynamics by extracting features from growth and pH curves, and its application to compare results between fecal communities from two healthy donors. Representative curves of (**a**) growth (OD_{600}) and (**b**) pH display features extracted from kinetic data fitted with smoothing splines. Letters in (**a**,**b**) correspond to features shown in panels (**c**-**n**). (**c**-**n**) Comparison of fermentation features by fecal communities from two healthy donors grown in triplicate on 5 g l^{-1} of each 653 SGs or 110 reference glycans as a sole carbohydrate source in MM29 medium. Correlation coefficients between two communities are shown. AUC of (**c**) growth and (**d**) pH data. (**e**) Difference between final and minimum pH. (**f**) Difference between maximum and final cell density. (**g**) Maximum acidification rate. (**h**) Maximum basification rate. (**i**) Maximum OD_{600}. (**j**) Maximum pH. (**k**) Minimum pH. (**l**) Growth lag measured where the tangent line to max growth rate intersects the starting cell density. (**m**) Maximum growth rate measured as maximum derivative of the growth curve. (**n**) Time of minimum pH. Values in (**c**-**n**) are shown are Z-scores calculated by subtracting the mean value for all compounds within a community and then dividing by the standard deviation of all compounds within a community. AUC was calculated using the trapezoidal rule. SG, Synthetic Glycan; OD_{600}, optical density at 600 nm; AUC, area under curve.

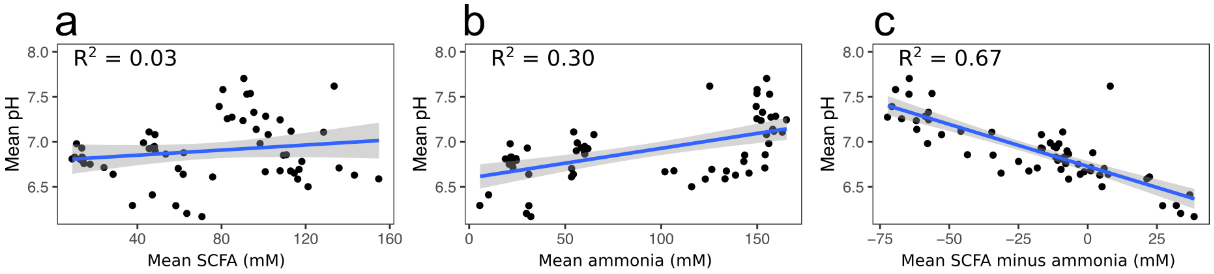

Supplementary Fig 2 Correlations between glycan culture pH and (**a**) SCFA production, (**b**) ammonia production, and (**c**) relative production of SCFAs and ammonia. Glycan fecal cultures were sampled for pH, SCFAs, and ammonia at 0,5,10,24, and 45h following inoculation. SCFA concentrations are the sum of acetate, propionate, and butyrate measured by gas chromatography with a flame ionization detector. Ammonia was measured by colorimetry (Abcam AB83360). Data points show means of triplicate cultures growing on 5 g l^{-1} of 12 randomly selected SGs in MM29 medium. Correlation lines and coefficients (R^2) are shown on each plot with error bands showing 95% confidence interval of the linear model. SG, Synthetic Glycan; SCFA, short-chain fatty acid.

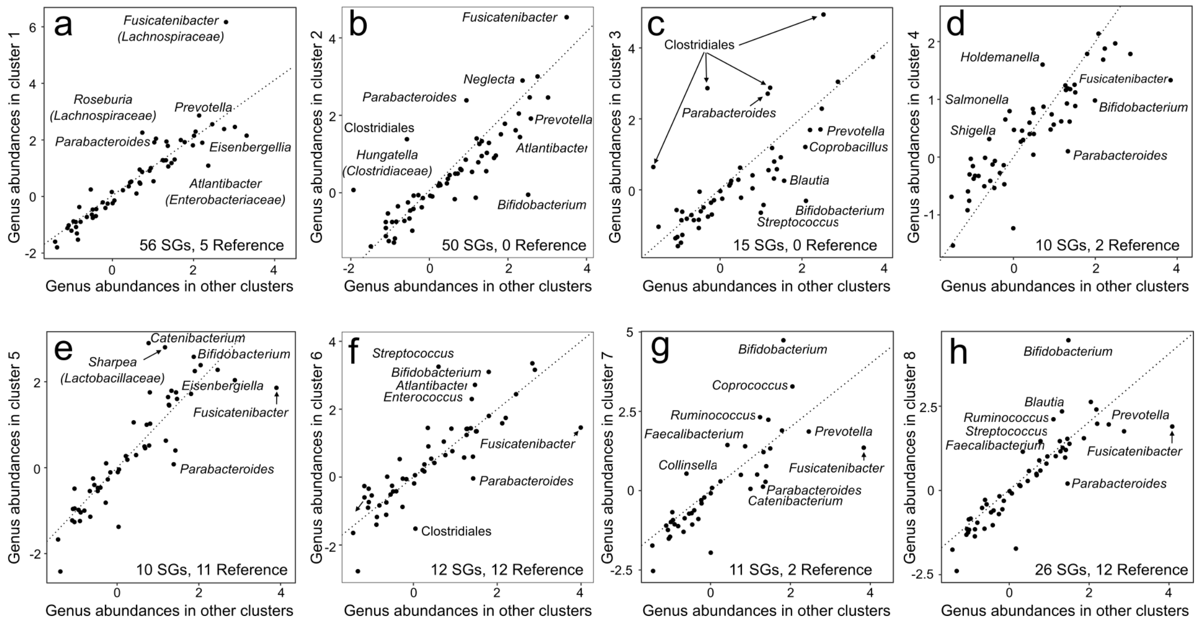

Supplementary Fig 3 Glycan fermentation differentially shifts taxonomic compositions of fecal cultures. As shown in Fig 2G, glycan cultures were classified into eight K-means clusters based on species-level mapping of metagenomic sequencing reads with BRF in cluster 1 and BQM in cluster 2. (**a-h**) Comparison of the abundance of each genus averaged across all glycans in the target cluster (y-axis) versus the abundance of that genus in the seven other clusters (x-axis). Genus abundances were calculated as log_2(percent reads mapping to the genus in a glycan culture/percent reads mapping to the genus in the no-glycan control culture). SG, Synthetic Glycan.

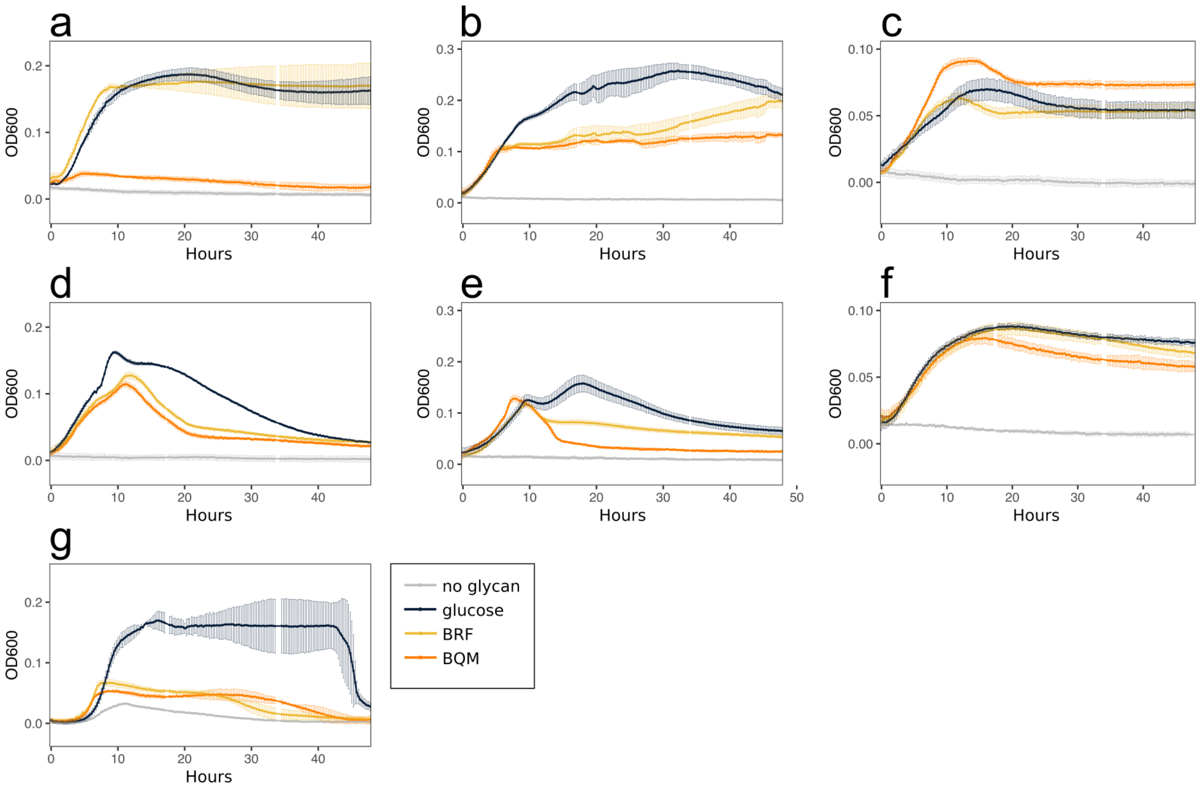

Supplementary Fig 4 Growth of phylogenetically diverse gut bacteria on BRF, BQM or glucose as a sole carbohydrate source in defined medium. Cultures of (**a**) *Bifidobacterium longum* subsp. *longum* ATCC 15707, (**b**) *Bacteroides uniformis* ATCC 8492, (**c**) *Blautia hansenii* ATCC 27752, (**d**) *Parabacteroides distasonis* ATCC 8503, (**e**) *Parabacteroides merdae* DMSZ 19495, (**f**) *Collinsella aerofaciens* ATCC 35085, and (**g**) *Clostridium difficile* ATCC BAA-1382 were grown in CM3 medium with 5 g l^{-1} of either BRF (yellow), BQM (orange), glucose (indigo) or without glycan supplementation (gray). Plots show mean cell density (OD_{600}) of triplicate cultures ±SD. SG, Synthetic Glycan; OD_{600}, optical density at 600 nm; SD, standard deviation.

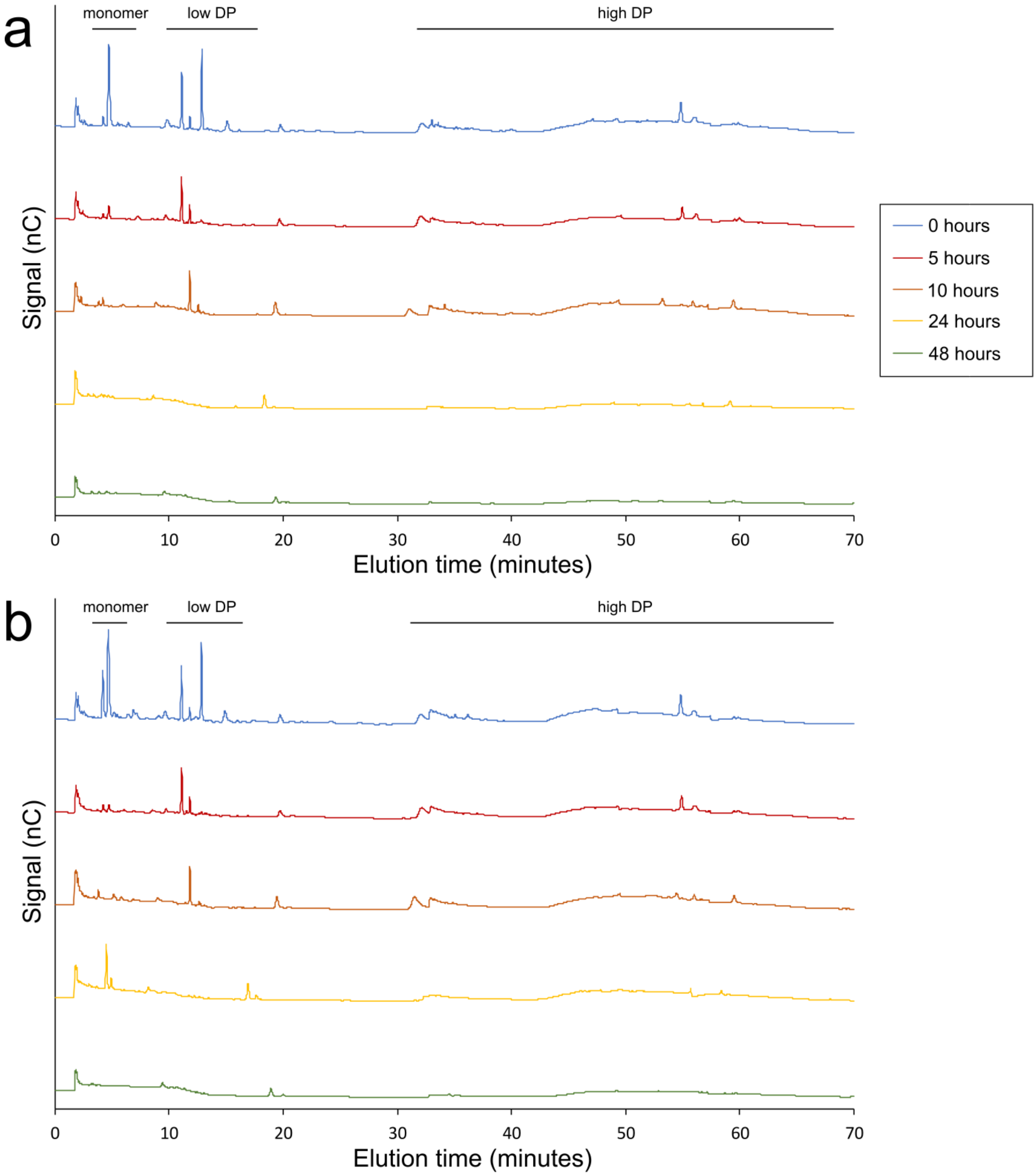

Supplementary Fig 5 Chromatographic fingerprints showing consumption of (**a**) BRF and (**b**) BQM during fermentation by fecal cultures as measured by HPAEC-PAD. Cultures were grown at Prodigest (Ghent, Belgium) in SHIME medium (ProDigest) containing 5 g L^{-1} SG for 48 hours at 37°C under anaerobic conditions. Glycan fingerprints after different incubation times (0, 5, 10, 24, 48 hours) are plotted as the detected signal (nC) versus the elution time (minutes). Elution periods corresponding to the glycan monomer, low DP fraction, and high DP fraction are shown above plots. HPAEC-PAD, high performance anion exchange chromatography with pulsed amperometric detection; nC, signal response, DP, degree of polymerization.

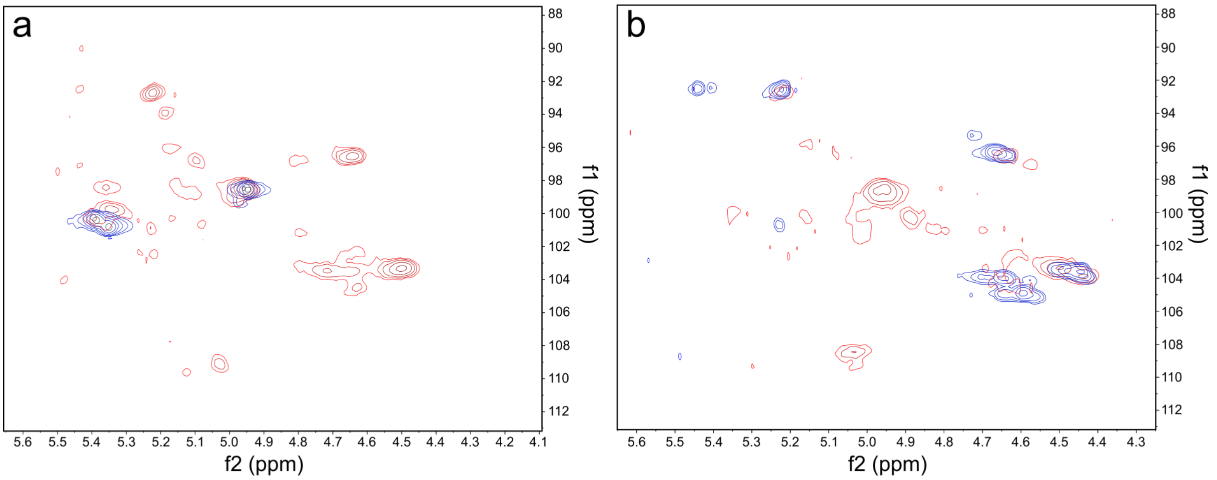

Supplementary Fig 6 Comparison of SG and reference glycan glycosidic linkages. ^{1}H-^{13}C HSQC 2D-NMR spectra of the anomeric regions show that the stereochemistry and regiochemistry of glycosidic linkages are distinct and more numerous in SGs (red) versus reference glycans (blue). (**a**) BRF (100% glucose) versus pullulan. (**b**) BQM (glucose-galactose) versus GOS. Axes are ^{1}H along the f2 (horizontal) axis and ^{13}C along the f1 (vertical) axis. SG, Synthetic Glycan; HSQC, heteronuclear single quantum coherence; 2D-NMR, two dimensional nuclear magnetic resonance spectroscopy.

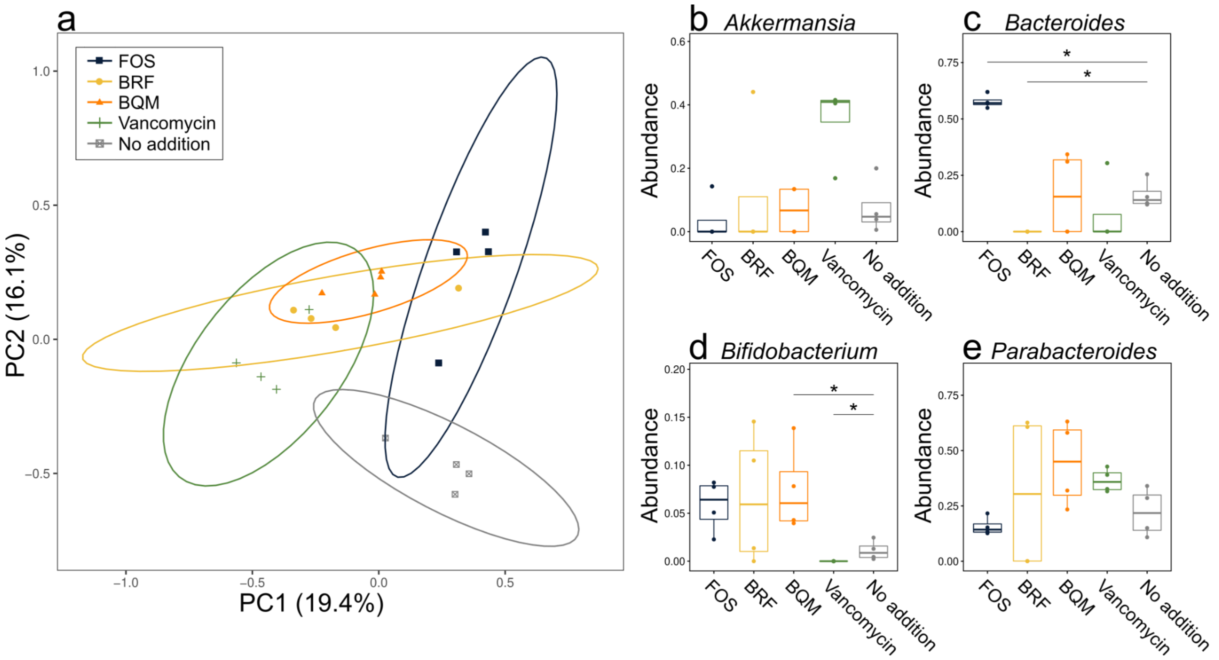

Supplementary Fig 7 Glycan treatment changes the taxonomic composition of the mouse gut microbiome based on fecal 16S metagenomics in a *C. difficile* infection model. Treatment groups (12 mice per group): no glycan (gray), vancomycin (green), FOS (indigo), BRF (yellow) and BQM (orange). Metagenomics shows genus-level read mapping from fecal samples 6 days after *C. difficile* infection with each data point representing the mean of a cage with 3 mice. (**a**) PCoA of metagenomic data calculated using a matrix of Bray-Curtis dissimilarities shows divergent microbiome compositions across treatments. Axes show percent variance explained by each PC. Ellipses are 95% confidence intervals. (**b-e**) Comparison of relative abundances of genera based on percent mapped reads for (**b**) *Akkermansia*, (**c**) *Bacteroides*, (**d**) *Bifidobacterium*, and (**e**) *Parabacteroides*. (**b-e**) Box plots show median and interquartile range; data points show individual cages of 3 mice. Asterisks show significance (*$p<0.05$) by two-sided Wilcoxon rank-sum test. PCoA, principal coordinates analysis; PC, principal coordinate.

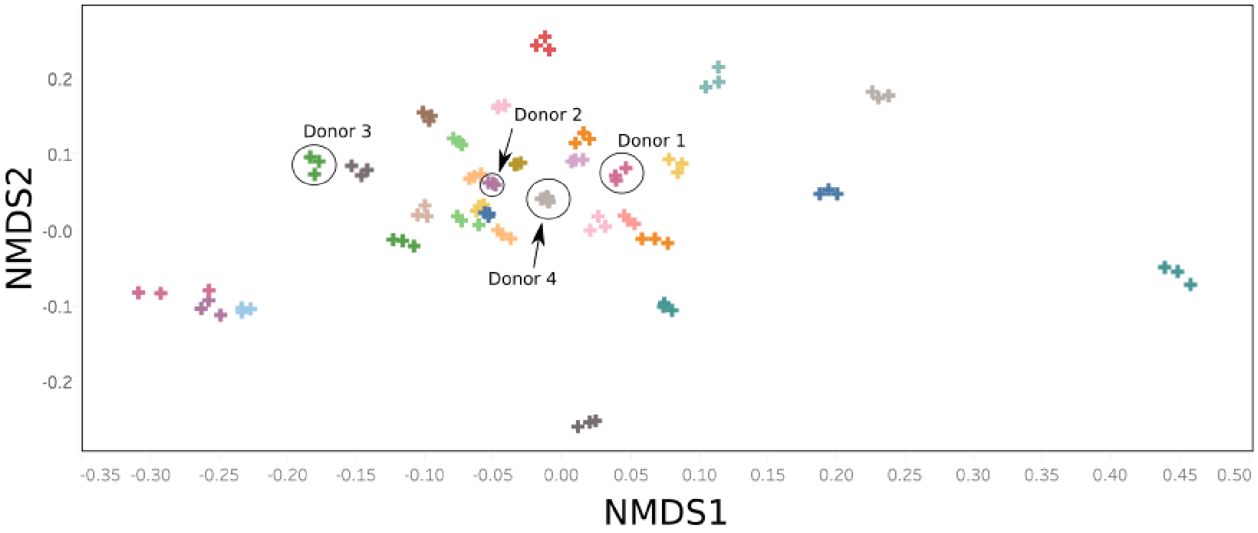

Supplementary Fig 8 Non-metric multidimensional scaling (NMDS) of metagenomic data calculated based on a matrix of Bray-Curtis dissimilarities using species-level mapping of sequencing reads from fecal samples collected from 32 healthy donors. Each sample was sequenced in triplicate with colors showing different donors. Donor 1-4 samples used in this study are circled.

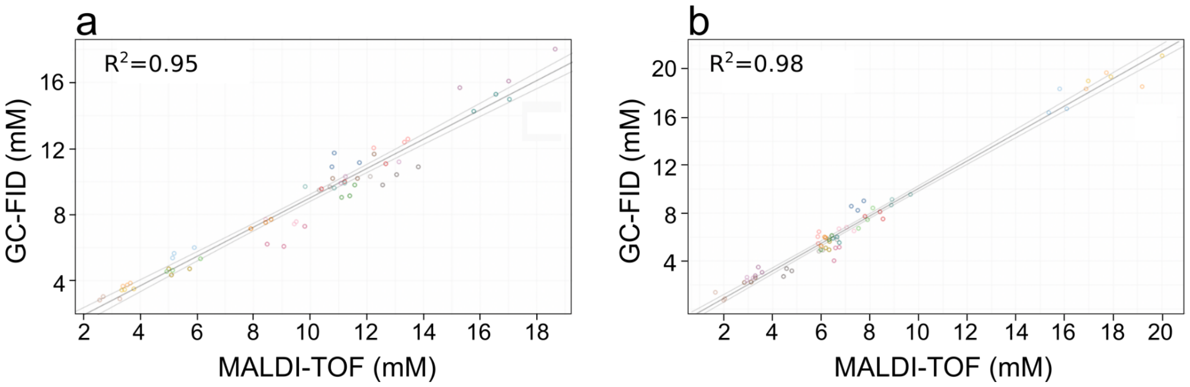

Supplementary Fig 9 Correlations between concentrations (mM) of (**a**) propionate and (**b**) butyrate in *ex vivo* fecal cultures measured by GC-FID and MALDI-TOF. Plots show triplicate fecal cultures fermenting each of 20 SGs in CM3 medium along with correlation coefficients (R^2). GC-FID, gas chromatography with flame ionization detector; MALDI-TOF, matrix-assisted laser desorption/ionization time-of-flight; SG, synthetic glycan.

Supplementary Table 1 Monosaccharide abbreviation codes.

Monosaccharide_abbreviation	Monosaccharide_name
glu	D-glucose
gal	D-galactose
man	D-mannose
lara	L-arabinose
fru	D-fructose
xyl	D-xylose
tre	D-trehalose
xylol	xylitol
gluol	D-glucitol
sorb	D-sorbitol
ery	erythritol
ino	myo-inositol
lfuc	L-fucose
rha	L-rhamnose
glua	D-glucuronic acid
manol	D-mannitol
galol	D-galactitol
glunac	N-acetyl-d-glucosamine
gala	D-galacturonic acid
rib	D-ribose

Supplementary Table 2 *Klebsiella pneumoniae, Escherichia coli,* and *Enterococcus faecium* strains used in fecal community spike-in and single strain growth assays. Table includes strain name, antibiotic resistance, and source.

Strain	Antibiotic resistance	Source
Enterococcus faecium ATCC 700221	Vancomycin and teicoplanin	ATCC
Enterococcus faecium Trans pharm 1006-Efcm = EF4-3525	Vancomycin	Transpharm
Enterococcus faecium TX0082	Ampicillin, erythromycin, kanamycin and vancomycin	Cesar A. Arias, MD. MSc, PhD
Enterococcus faecium ERV107	Vancomycin	Cesar A. Arias, MD. MSc, PhD
Enterococcus faecium ERV99	Vancomycin	Cesar A. Arias, MD. MSc, PhD
Enterococcus faecium TX1330	none	Cesar A. Arias, MD. MSc, PhD
Escherichia coli ATCC BAA-97	none	ATCC
Escherichia coli ATCC BAA-2340	multi-drug resistance carbapenemase	ATCC
Escherichia coli CRE strain CDC 001 SAMN04014842	Carbapenem	CDC
Escherichia coli CRE 32765	Carbapenem	Barry Kreiswirth
Escherichia coli CRE 36415	Carbapenem	Barry Kreiswirth
Escherichia coli CRE 34398	Carbapenem	Barry Kreiswirth
Klebsiella pneumoniae CRE strain CDC 003 SAMN04014844	Carbapenem	CDC
Klebsiella pneumoniae ATCC 33259	none	ATCC
Klebsiella pneumoniae ATCC BAA-1705	multi-drug resistance carbapenemase	ATCC
Klebsiella pneumoniae CRE 48701	Carbapenem	Barry Kreiswirth
Klebsiella pneumoniae CRE 49450	Carbapenem	Barry Kreiswirth
Klebsiella pneumoniae CRE 49434	Carbapenem	Barry Kreiswirth

Supplementary Table 3 Mega Medium 29 (MM29) recipe

Compound	Final Concentration
Tryptone Peptone	10g/L
Yeast Extract	5g/L
L-Cysteine	0.5g
1M Potassium Phosphate, pH 7.2	100ml/L
TYG Salts Solution	40ml/L
Vitamin K	1 mg/L
Calcium chloride ($CaCl_2$) (0.8% stock)	1ml/L
Iron sulfate ($FeSO_4 \cdot 7H_2O$) (0.4mg/mL stock)	1ml/L
Resazurin (0.25mg/mL stock)	4ml/L
Histidine/Hematin (0.2M/2mM stock)	1ml/L
Tween 80 (25% stock)	2ml/L
Meat Extract	5g/L
Trace mineral supplement (MD-TMS purchased from ATCC)	10ml/L
Vitamin supplement (MD-VS purchased from ATCC)	10ml/L
Acetate	1.7 ml/L

1. Bring final volume to 1L
2. Bring to pH 7 by adding base (e.g. NaOH)
3. Filter sterilize media with a 0.2um filter system (wrapped in foil) and store protected from light at 4°C (wrap in foil)
4. Add glycan carbohydrate source (5 g/L final concentration), as appropriate

TYG salts solution

Components	Concentration
Magnesium sulfate ($MgSO_4 \cdot 7H_2O$)	0.5 g/L
Sodium bicarbonate ($NaHCO_3$)	10.0 g/L
Sodium chloride (NaCl)	2.0 g/L

Supplementary Table 4 Clostridia Minimal 3 (CM3) medium recipe

Compound	Final Concentration (mg/L)
Sodium chloride (NaCl)	900
Calcium chloride (CaCl2-2H2O)	26
Magnesium chloride (MgCl2-6H2O)	20
Manganese chloride (MnCl2-4H2O)	10
Ammonium sulfate ((NH4)2SO4)	40
Iron sulfate (FeSO4-7H2O)	4
Cobalt chloride (CoCl2-6H2O)	1
Potassium phosphate dibasic (KH2PO4)	300
Disodium phosphate (Na2HPO4)	1500
Sodium bicarbonate (NaHCO3)	5000
Biotin (vitamin B7)	0.125
Pyridoxine (vitamin B6)	1
Pantothenate (vitamin B5)	1
Histidine	75
Glycine	75
Tryptophan	75
Arginine	150
Methionine	150
Threonine	150
Valine	225
Isoleucine	225
Leucine	300
Cysteine	400
Proline	450

1. Bring final volume to 1L
2. Check pH (~7.7 - 8) and record filter sterilize using a bottle/filter unit with a 0.2 micron filter and store at 4°C.
3. Filter sterilize media with a 0.2um filter system and store protected at 4°C
4. Add glycan carbohydrate source (5 g/L final concentration), as appropriate

Supplementary Table 5 Scoring systems for stool consistency in mouse DSS colitis study.

Stool Score	Description
0	normal, well-formed pellet
1	loose stool, soft and retains shape
2	loose stool, abnormal with excessive moisture
3	watery and diarrhea
4	bloody diarrhea

Supplementary Table 6 Scoring systems for endoscopies in mouse DSS colitis study.

Endoscopy Score	Description
0	Normal
1	Loss of vascularity
2	loss of vascularity and friability
3	friability and erosions
4	ulcerations and bleeding

Supplementary Table 7 Histopathologic grading of colitis in mouse DSS colitis study. GC, goblet cells; Inflm, inflammation; LP, lamina propria; macs, macrophages; S/M, submucosa.

**Category**	**Score**			
	**1**	**2**	**3**	**4**
**Inflammation**	Mild, focal or widely separated multifocal inflm in LP	Moderate, multifocal extending into s/m or segmental mucosal inflm	Marked, multifocal or locally extensive inflm (large numbers of neutrophils, macs) involving the s/m, + hemorrhage	Severe, diffuse inflm (large numbers of neutrophils, macs), transmural, + hemorrhage
**Crypt Loss**	1-25% area affected	26-50% area affected	51-75% area affected	76-100% area affected
**LP fibrosis**	1-25% area affected	26-50% area affected	51-75% area affected	76-100% area affected
**Edema**	Multifocal and mucosal (mild)	Segmental mucosal + S/M involvement (moderate) or multifocal S/M	Segmental mucosal with S/M involvement (marked)	Diffuse and transmural (severe)
**Erosion/ulcer**	Focal/multifocal erosion	Multifocal/segmental erosion or focal ulcer	Diffuse erosion or multifocal/segmental ulcer	Diffuse and transmural ulcer
**Hyperplasia/ dysplasia**	2x normal crypts with no GC loss	2x-3x normal crypts with mild GC loss	>4x normal crypts, marked GC loss	>4x normal crypts, complete GC loss with crypt arborization

Supplementary Table 8 Scoring of disease severity in mouse *C. difficile* infection study.

Score	Description
0	Normal
1	Lethargic
2	Lethargic and hunched
3	Lethargic, hunched, and wet tail/abdomen
4	Lethargic, hunched, wet tail/abdomen, and hypothermic
5	Dead
