# Supplementary material for: Synthetic glycans control gut microbiome structure and mitigate colitis in mice
Source: Nat Commun. 2022 Mar 10;13:1244. doi: 10.1038/s41467-022-28856-x (PMC8913648; doi:10.1038/s41467-022-28856-x)
Supplement: Supplementary file 2 — Description of Additional Supplementary Files [file 41467_2022_28856_MOESM2_ESM.docx]

**Description of Supplementary Data files for “Synthetic glycans control gut microbiome structure and mitigate colitis in mice”**

**Supplementary Data 1** Glycans included in growth and pH fermentation dynamics assays (653 SGs, 110 reference glycans). Table includes compound name, compound type (SG or reference glycan), monosaccharide composition, purity (percent compound with degree of polymerization greater than purity threshold), purity threshold, source, and fermentation dynamics group as shown in Fig 1. Dendrogram cluster compositions: Group 1 (49 SGs, 57 reference glycans), Group 2 (77 SGs, 6 reference glycans, no-glycan control), Group 3 (94 SGs), Group 4 (8 SGs, 23 reference glycans), Group 5 (425 SGs, 24 reference glycans). SG, Synthetic glycan; degree of polymerization, DP; no data, ND. Monosaccharide abbreviations are defined in Supplementary Table 1.

**Supplementary Data 2** Glycans included in assays of gas production by fecal communities. Table includes compound type (SG or reference glycan), monosaccharide composition, source, and fermentation dynamics group as defined in Fig 1. SG, Synthetic Glycan, ND, no data. Monosaccharide abbreviations are defined in Supplementary Table 1.

**Supplementary Data 3** Glycans included in metagenomic sequencing of fecal cultures. Table includes compound name, compound type (SG or reference glycan), monosaccharide composition, purity (percent compound with degree of polymerization greater than purity threshold), purity threshold, source, and sequencing cluster as shown in Fig 2G. Abbreviations: SG, Synthetic Glycan, No data, ND; Degree of polymerization, DP. Monosaccharide abbreviations are defined in Supplementary Table 1.

**Supplementary Data 4** Glycans included in single-strain growth assays of *Klebsiella pneumoniae, Escherichia coli*, and *Enterococcus* *faecium*. Table includes compound name, type (SG or reference glycan), monosaccharide composition, purity (percent compound with DP greater than purity threshold), purity threshold, and source. SG, Synthetic Glycan; DP, degree of polymerization; ND, no data. Monosaccharide abbreviations are defined in Supplementary Table 1.

**Supplementary Data 5** Glycans included in pathogen spike-ins of fecal communities from a healthy subject. Table includes compound name, type (SG or reference glycan), monosaccharide composition, purity (percent compound with DP greater than purity threshold), purity threshold, and source. SG, Synthetic Glycan; DP, degree of polymerization; ND, no data. Monosaccharide abbreviations are defined in Supplementary Table 1.
