# Supplementary material for: Synthetic glycans control gut microbiome structure and mitigate colitis in mice
Source: Nat Commun. 2022 Mar 10;13:1244. doi: 10.1038/s41467-022-28856-x (PMC8913648; doi:10.1038/s41467-022-28856-x)
Supplement: Supplementary file 8 — Reporting Summary [file 41467_2022_28856_MOESM8_ESM.pdf]

nature portfolio

Corresponding author(s): Andrew C Tolonen, Johan van Hylckama Vlieg

Last updated by author(s): Feb 1, 2022

Reporting Summary

Nature Portfolio wishes to improve the reproducibility of the work that we publish. This form provides structure for consistency and transparency in reporting. For further information on Nature Portfolio policies, see our Editorial Policies and the Editorial Policy Checklist.

Statistics

For all statistical analyses, confirm that the following items are present in the figure legend, table legend, main text, or Methods section.

n/a	Confirmed	
[ ]	[x]	The exact sample size (n) for each experimental group/condition, given as a discrete number and unit of measurement
[ ]	[x]	A statement on whether measurements were taken from distinct samples or whether the same sample was measured repeatedly
[ ]	[x]	The statistical test(s) used AND whether they are one- or two-sided<br>*Only common tests should be described solely by name; describe more complex techniques in the Methods section.*
[ ]	[x]	A description of all covariates tested
[ ]	[x]	A description of any assumptions or corrections, such as tests of normality and adjustment for multiple comparisons
[ ]	[x]	A full description of the statistical parameters including central tendency (e.g. means) or other basic estimates (e.g. regression coefficient) AND variation (e.g. standard deviation) or associated estimates of uncertainty (e.g. confidence intervals)
[ ]	[x]	For null hypothesis testing, the test statistic (e.g. F, t, r) with confidence intervals, effect sizes, degrees of freedom and P value noted<br>*Give P values as exact values whenever suitable.*
[x]	[ ]	For Bayesian analysis, information on the choice of priors and Markov chain Monte Carlo settings
[x]	[ ]	For hierarchical and complex designs, identification of the appropriate level for tests and full reporting of outcomes
[ ]	[x]	Estimates of effect sizes (e.g. Cohen's d, Pearson's r), indicating how they were calculated

Our web collection on statistics for biologists contains articles on many of the points above.

Software and code

Policy information about availability of computer code

Data collection	The methods and code for the R package phgrofit (version 1.0.2) to extract physiological descriptors from the kinetic pH and OD 600 data are freely available on Github: doi: 10.5281/zenodo.5948925
Data analysis	Statistical analyses were performed using R software. The vegan R package 59 (version 2.5-6) was used to compute the Shannon diversity, Bray-Curtis dissimilarity, and multidimensional scaling measures and coordinates. Size exclusion chromatography data was analyzed using Agilent Cirrus GPC/SEC software (version 3.4.2). xCGE-LIF data were analyzed with glyXtoolCE™ (glyXera) glycoanalysis software to perform migration time alignment, raw data smoothing, DP-range specific interval picking, and normalization of total peak areas. NMR spectra were analyzed using spectra were analyzed using the MestReNova software (version: 12.0.0-20080) from Mestrelab Research. MALDI-TOF data was analyzed using Bruker PharmaPulse 2.1 software.

For manuscripts utilizing custom algorithms or software that are central to the research but not yet described in published literature, software must be made available to editors and reviewers. We strongly encourage code deposition in a community repository (e.g. GitHub). See the Nature Portfolio guidelines for submitting code & software for further information.

Data

Policy information about availability of data

All manuscripts must include a data availability statement. This statement should provide the following information, where applicable:

- Accession codes, unique identifiers, or web links for publicly available datasets
- A description of any restrictions on data availability
- For clinical datasets or third party data, please ensure that the statement adheres to our policy

The DNA sequencing reads for this study are available in the NCBI SRA database as project accessions PRJNA800419, PRJNA800763, PRJNA800792, and PRJNA800405. Supporting data are available in Supplementary Information, Supplementary Datasets, and the Source data file. All requests will be reviewed by Kaleido Biosciences to verify whether the request is subject to any intellectual property or confidentiality obligations.

Field-specific reporting

Please select the one below that is the best fit for your research. If you are not sure, read the appropriate sections before making your selection.

[x] Life sciences [ ] Behavioural & social sciences [ ] Ecological, evolutionary & environmental sciences

For a reference copy of the document with all sections, see nature.com/documents/nr-reporting-summary-flat.pdf

Life sciences study design

All studies must disclose on these points even when the disclosure is negative.

Sample size	Microbial cultures were grown and analyzed in triplicate for all experiments. Eight animals were included per treatment in the DSS colitis mouse study and twelve animals per treatment were included in the C. difficile infection mouse study.
Data exclusions	No data was excluded from this study.
Replication	Microbial experiments were replicated at least once with all attempts at replication being successful. Mouse experiments were performed once.
Randomization	Mice were randomly allocated into treatment groups for all studies.
Blinding	Since the same researchers performing treatments were also responsible for planning and analysis of experiments, they were not blinded to allocation during experiments or outcome assessments.

Reporting for specific materials, systems and methods

We require information from authors about some types of materials, experimental systems and methods used in many studies. Here, indicate whether each material, system or method listed is relevant to your study. If you are not sure if a list item applies to your research, read the appropriate section before selecting a response.

Materials & experimental systems

n/a	Involved in the study	
[x]	[ ]	Antibodies
[x]	[ ]	Eukaryotic cell lines
[x]	[ ]	Palaeontology and archaeology
[ ]	[x]	Animals and other organisms
[x]	[ ]	Human research participants
[x]	[ ]	Clinical data
[x]	[ ]	Dual use research of concern

Methods

n/a	Involved in the study	
[x]	[ ]	ChIP-seq
[x]	[ ]	Flow cytometry
[x]	[ ]	MRI-based neuroimaging

Animals and other organisms

Policy information about studies involving animals; ARRIVE guidelines recommended for reporting animal research

Laboratory animals	In the DSS colitis study, male C57Bl/6 mice (Taconic Biosciences) at 6-8 weeks of age were randomized into cages each containing a treatment group of eight mice at Biomodels LLC (Waltham MA, USA). Mice were kept in rooms provided with filtered air at 65-75 °F with 30-70% relative humidity. Rooms were on a 12h:12h light:dark cycle with no twilight and a minimum of 12-15 air changes per hour. Animals were fed diet 5053 (Lab Supply, Fort Worth TX, USA) throughout the study with water provided ad libitum.

	In the C. difficile infection study, female C57Bl/6 mice (Harlan Laboratories) weighing 16-18g were randomly allocated into treatment groups of 12 animals (3 animals per cage) at Trans Pharm Pre-clinical Solutions (Jackson MI, USA). Animal room conditions were the same as in the DSS colitis study. Mice were fed Teklad Global Rodent Diet 2918 (Harlan) and water ad libitum.
Wild animals	No wild animals were used in this study.
Field-collected samples	No field collected samples were used in this study.
Ethics oversight	Informed consent was obtained from all donors before fecal sample collection. Mouse studies were performed in laboratories accredited by the Association for Assessment and Accreditation of Laboratory Animal Care (AAALAC) at either TransPharm or Biomodels LLC.

Note that full information on the approval of the study protocol must also be provided in the manuscript.
